# Exploring the mechanism of Heidihuang Pill in the treatment of osteoporosis based on network pharmacology, molecular docking, and experimental validation

**DOI:** 10.3389/fendo.2025.1664254

**Published:** 2025-12-15

**Authors:** Zenghui Tian, Kaiying Cui, Yuxiao Tian, Yungang Chen, Guoyan Liu, Farong Zhang, Yanke Hao, Yingying Li

**Affiliations:** 1 College of First Clinical Medical, Shandong University of Traditional Chinese Medicine, Jinan, China; 2 Affiliated Hospital of Shandong University of Traditional Chinese Medicine, Jinan, China; 3 College of Second Clinical Medical, Shandong University of Traditional Chinese Medicine, Jinan, China

**Keywords:** Heidihuang Pill, osteoporosis, AGE-RAGE signaling pathway, HIF-1 signaling pathway, network pharmacology

## Abstract

**Background:**

Currently, anti-osteoporosis (OP) treatment imposes a certain economic burden on patients and society; therefore, studies have focused on exploring anti-OP therapies. As a classic prescription in traditional Chinese medicine (TCM), the Heidihuang Pill (HP) was clinically shown to alleviate OP. However, the mechanism behind its effect remains unclear. We aimed to determine this mechanism using methods such as network pharmacology and experimental verification.

**Methods:**

Network pharmacology was used to identify the active ingredients, targets, and mechanisms of action of HP in treating OP. Molecular docking technology was then used to verify the interactions between the active ingredients and target proteins. Finally, an OP rat model was established through *in vivo* experiments to validate the results obtained from network pharmacology.

**Results:**

Overall, 178 targets were retrieved, which are key targets of HP for treating OP. Protein-Protein Interaction network analysis showed that RAC-alpha serine/threonine-protein kinase (Akt1) (degree =132) was the most reliable target of HP for treating OP. Gene Ontology functional analysis revealed that the regulation of HP on OP mainly occurs as follows: it mainly manifests as a response to hormones in biological processes; it mainly acts on membrane rafts in cellular components; and it mainly involves the binding of transcription factors in molecular functions. Kyoto Encyclopedia of Genes and Genomes pathway enrichment analysis indicated that the Advanced Glycation End products/Receptor for Advanced Glycation End products (AGE-RAGE) signaling pathway in diabetic complications and the hypoxia-inducible factor (HIF)-1 signaling pathway are the main pathways involved in the HP treatment of OP. Molecular docking results showed that Akt1 has a good binding ability with peoniflorin, with a binding energy of -56.66 kcal/mol. *In vivo* experiments confirmed that the trabecular bone mineral density, trabecular number, and bone volume fraction of model rats treated with HP significantly increased (*P* < 0.05 vs. the model group). Histopathological staining showed that the number and morphology of the trabecular bones improved (vs. the model group). Additionally, the expression of HIF-1 signaling protein in rat bone tissue increased, while the expression of AGE/RAGE signaling protein decreased (*P* < 0.05, vs. the model group).

**Conclusion:**

Peoniflorin, the main active ingredient in HP, acts on Akt1 and treats OP through the AGE-RAGE and HIF-1 signaling pathways. This study showed the precise molecular pathways and core therapeutic targets of HP in treating OP, and provides a scientific basis and new research direction for treating OP with TCM.

## 1 Introduction

Osteoporosis (OP) is the most common metabolic bone disease and is characterized by the loss of bone strength caused by an imbalance in bone homeostasis, leading to increased bone fragility and fracture risk (1). With an aging global population, the number of individuals affected by OP continually increases. Studies have shown that 10.2% of adults over 50 years of age have OP, with a likely projection of 13.6% by 2030 (2). Currently, OP and its related fractures have become common causes of mortality among older adults (3). The primary treatment for OP involves the systemic administration of medications to reduce bone resorption or stimulate bone formation, thereby preventing fractures. Examples of these medications include anti-resorptive drugs, including bisphosphonates and denosumab, bone-forming agents such as teriparatide, and dual-action drugs that stimulate bone formation and inhibit resorption, such as romosozumab (4). However, as clinical research progresses, it has been recognized that anti-OP treatment requires a prolonged process. Long-term use of these medications may result in adverse events, including osteonecrosis of the jaw, atrial fibrillation, fever, and myalgia (5). Additionally, the prolonged use of medications and healthcare management for OP imposes significant economic burdens on patients and society (6). In Europe, OP has become a major and growing healthcare challenge, with annual medical costs exceeding €56 billion (7). Consequently, exploring effective and cost-efficient anti-OP therapies remains a critical research focus (8).

Traditional Chinese medicine (TCM) has a long history of preventing and treating OP, holding that “deficiency of the spleen and kidney” is the primary pathogenesis of OP (9). Therefore, in treating OP with TCM, tonifying the spleen and kidney is also key. As a classic prescription passed down in TCM, Heidihuang Pill (HP) is composed of Rehmanniae (熟地黄), Atractylodes (苍术), Dried ginger (干姜), and jujube (大枣), with the efficacy of tonifying the kidney and spleen. Modern pharmacological studies have shown that the aqueous extract of Rehmanniae improves bone health and alleviates bone microstructure damage caused by OP by regulating the miR-29a-3p/NFIA/Wnt signaling axis (10). Extracts of Atractylodes (including water and ethanol extracts) enhance osteogenesis and inhibit osteoclast formation (11). Zingiberone, a component of dried ginger, enhances ferroptosis sensitivity and inhibits osteoclast formation. It also promotes bone remodeling to improve OP (12). ZJMP-2, a water-soluble polysaccharide component of Jujubae, increases the number of bone marrow cells (13). Moreover, clinical applications discovered that HP can improve OP by regulating calcium and phosphorus metabolism and increasing bone mineral density (BMD) and bone volume fraction (BV/TV) (14).

Currently, studies on HP have mainly focused on its role in improving kidney disease (15, 16); however, the mechanism by which it improves OP remains unclear. Therefore, we are the first to explore this mechanism using methods such as network pharmacology and experimental verification. This study showed the precise molecular pathways and core therapeutic targets of HP for treating OP, and provides a scientific basis and new research direction for treating OP with TCM.

## 2 Materials and methods

### 2.1 Network pharmacology

#### 2.1.1 Screening of active components and target proteins of Heidihuang Pill

The components of HP were identified using the keywords “shudihuang,” “cangzhu,” “ganjiang,” and “dazhao” across three databases: Traditional Chinese Medicine Systems Pharmacology (TCMSP) (http://tcmspw.com/tcmsp.php, accessed: 2025-03) (17), Integrative Pharmacology-based Research Platform of Traditional Chinese Medicine (http://www.tcmip.cn/TCMIP/index.php, accessed: 2025-03) (18), and A Bioinformatics Analysis Tool for Molecular Mechanism of Traditional Chinese Medicine (http://bionet.ncpsb.org.cn/batman-tcm/, accessed: 2025-03) (19) databases. Active ingredients were screened using pharmacokinetic core parameters (absorption, distribution, metabolism, excretion (ADME)): oral bioavail-ability (OB) ≥30% and drug similarity (DL) ≥0.18 (8). The corresponding target protein of each active ingredient was then obtained from the TCMSP database. Finally, the UniProt database (https://www.uniprot.org/, accessed: 2025-03) was used to unify the names of the target proteins. Duplicate items were removed, and the data were merged and converted into gene symbols.

#### 2.1.2 Screening of osteoporosis-related target proteins

Relevant targets for OP were identified by searching the GeneCards (http://www.genecards.org, accessed: 2025-03), OMIM (http://www.omim.org, accessed: 2025-03), and DrugBank (https://go.drugbank.com, accessed: 2025-03) databases with the keyword “Osteoporosis”. Subsequently, the target data were standardized by unifying protein names, deduplicating entries, merging datasets, and converting gene symbols through the UniProt database.

#### 2.1.3 Construction and analysis of the drug-active component-target-disease network

The Venny 2.1 system (https://bioinfogp.cnb.csic.es, accessed: 2025-03) was used to generate a Venn diagram to identify shared targets between HP active components and OP-related targets. Cytoscape 3.7.1 software (http://cytoscape.org) was used to map the relationships between drugs, active components, common targets, and OP, resulting in a drug-active component-target-disease network.

#### 2.1.4 Construction and analysis of the protein–protein interaction network

The shared HP-OP targets were uploaded to the STRING database (https://string-db.org, accessed: 2025-03) to construct a protein-protein interaction (PPI) network. The species was limited to Homo sapiens with a medium confidence interaction score threshold (>0.4). Subsequent topological analysis of the network was conducted using Cytoscape 3.7.1, and the CytoNCA plugin was used to calculate the node degree.

#### 2.1.5 Gene ontology functional enrichment analysis and kyoto encyclopedia of genes and genomes pathway enrichment analysis

Shared HP-OP targets were imported into Metascape (http://metascape.org, accessed: 2025-03) for functional enrichment analysis. Gene ontology (GO) terms (biological process, molecular function, and cellular component) and Kyoto encyclopedia of genes and genomes (KEGG) pathways were analyzed using Homo sapiens settings, and the results were visualized.

### 2.2 Molecular docking

Molecular docking analysis was conducted using Discovery Studio (DS) v3.5 (Biovia. San Diego, CA, USA). The crystal structures of the proteins (targets) were extracted from the Protein Data Bank (https://www.rcsb.org/, accessed: 2025-04). SDF files of the 3D structures of the active components were downloaded from the PubChem database (https://pubchem.ncbi.nlm.nih.gov/, accessed: 2025-04).

First, the ligand was imported in SDF format into the DS software. Under the “Small Molecules” window, “Prepare Ligands” was first conducted to prepare small molecules, with the ionization method based on a pH range of 6.5-8.5. Then, “Full Minimization” was conducted to optimize the small molecules, using the CHARMm force field as the additional force field, the Smart Minimizer as the algorithm, and setting the maximum number of steps to 2000. Second, the protein was imported in PDB format into the DS software. In the “Automatic Preparation” section under the “Macromolecules” window, “Prepare Protein” was selected to remove crystal water and calculate the CHARMm force field. Next, the active center was defined in the “Define and Edit Binding Site” window under “Receptor-Ligand Interactions” in the DS software. Then, the semi-flexible docking mode of Dock Ligands (CDOCKER) was selected in the software for molecular docking. Finally, redocking was conducted to evaluate the binding mode before formal molecular docking, and then Pymol 2.5.7 (Portland, OR, US) was used for visual analysis.

### 2.3 Experimental validation

#### 2.3.1 Animals and grouping

Twenty-four 3-month-old female Sprague-Dawley (SD) rats (body weight: 240–300 g) were purchased from Beijing Vital River Laboratory Animal Technology Co., Ltd. (license number: SCXK (Jing) 2021-0006). The rats were sheltered in the SPF-grade laboratory of the Animal Experiment Center of the Affiliated Hospital of Shandong University of Traditional Chinese Medicine. The environmental conditions included constant temperature and humidity (room temperature: 22–26 °C; relative humidity: 50 ± 5%, a 12-hour light/dark cycle, and ad libitum access to food and water).

According to the 3R principles for laboratory animals (20), 24 female SD rats were randomly divided into four groups (n = 6 per group) using a random number table: NC-Group, model, experimental, and positive control groups. The Experimental Animal Ethics Committee of the Shandong University of Traditional Chinese Medicine (approval number: SDSZYYAWE20231219001) approved this study protocol.

#### 2.3.2 Drugs

HP was composed of Rehmanniae (熟地黄, 32g), Atractylodes (苍术, 32g), Dried ginger (干姜, 2g), and jujube (大枣, 34g). Herbal pieces were purchased from the pharmacy of the Affiliated Hospital of Shandong University of TCM and authenticated by TCM pharmacists in the hospital. The preparation was conducted in a decoction room, and the four herbs were soaked in 10 volumes of water for 30 min. The mixture was decocted twice (2 h each time), filtered through a gauze, and the filtrates were combined. The resulting solution was at a final concentration of 1 g/mL and stored at 4 °C for later use.

Alendronate Sodium Tablets (Beijing Fuyuan Pharmaceutical Co., Ltd.; National Drug Approval No. H20059029) were used as positive controls.

#### 2.3.3 Major equipment and reagents

Micro-Computed Tomography (CT) (Model: NC-200), Multicolor Fluorescence Imaging System (Model: GE Amersham Imager 600), Embedding Machine (Model: JB-P5), Paraffin Microtome (Model: Leica RM2255), Automated Slide Analysis System (Model: HS6), and Bicinchoninic Acid (BCA) Protein Quantification Kit (Catalog No. P0010; Beyotime Biotechnology, Shanghai, China), Anti- hypoxia-inducible factor (HIF)-1α Antibody (cat. no. ab179483; Abcam, Cambridge, UK), anti-AGER/RAGE (cat. no. 83759-5-RR; Proteintech, USA), Anti- Glyceraldehyde-3-phosphate dehydrogenase (GAPDH) Antibody (cat. no. 10494-1-AP, Proteintech, USA), horseradish Peroxidase (HRP)-Labeled goat anti-rabbit IgG (cat. no. 20536-1-AP, Proteintech, USA).

#### 2.3.4 Animal modeling and drug administration

After one week of acclimatization, rats in the model, experimental, and positive control groups were anesthetized using an intraperitoneal injection of Zoletil (15 mg/kg) + Xylazine Hydrochloride (5 mg/kg). After successful anesthesia, the dorsal area was shaved and disinfected. A longitudinal incision (approximately 1.5 cm) was made below the ribs on both sides of the spine. The subcutaneous tissues and muscles were bluntly dissected to expose the ovaries. Both fallopian tubes were ligated, and bilateral ovariectomy was conducted to establish an ovariectomized OP model. Hemostasis, irrigation, and disinfection were completed, followed by layered suturing of the muscles and skin. Postoperatively, the rats received intramuscular penicillin (8×104U/rat) for 3 consecutive days to prevent infection.

Drug interventions commenced 7 days after modeling. Based on body surface area conversion (21), the experimental group received 10.43 g/kg/day of HP decoction through oral gavage. The positive control group was administered 7.35 mg/kg alendronate sodium (prepared by suspending finely ground tablets in purified water to 2 mg/mL, 3.675 mL/kg) once a week. The NC-Group and model groups received equivalent volumes of saline daily through oral gavage. All treatments were administered for 12 weeks.

#### 2.3.5 Sample collection

After gavage, the rats were euthanized by cervical dislocation. The hair of their bodies was shaved, and the entire body was disinfected by immersing the rats in 75% alcohol. The left and right femurs were then harvested. For the right femur, it was fixed with 4% paraformaldehyde fixative at 4 °C for over 48 h, followed by Micro-CT imaging analysis. Subsequently, the femur was decalcified with 10% EDTA decalcification solution at 4 °C for 4 weeks, and paraffin tissue sections were prepared. The left femur was immediately immersed in liquid nitrogen for quick freezing and stored at -80 °C for future use.

#### 2.3.6 Femoral micro-computed tomography analysis

The right femurs from each group was subjected to micro-CT scanning. Then, three-dimensional reconstruction was conducted using NRecon software (version 1.20.3.0; Bruker MicroCT, Belgium) on selected regions of interest. Quantitative analysis of bone microstructure parameters was conducted with CT Analyzer (version 1.20.3.0) under standardized thresholds. BMD, BV/TV, and trabecular number (Tb.N) were calculated.

#### 2.3.7 Femoral histological staining

Paraffin-embedded right femur samples were sectioned into 3.0–4.0 μm-thick slices using a microtome. The sections were stained with hematoxylin and eosin (H&E) per the manufacturer’s protocol. Histopathological evaluation of the trabecular bone morphology and quantity was conducted using a light microscope.

#### 2.3.8 Detection of hypoxia-inducible factor-1 and advanced glycation end products/receptor for advanced glycation end products protein expression in bone tissues by western blot

The left femurs of rats in each group were taken, sequentially crushed into a mortar, rapidly ground into a powder with multiple additions of liquid nitrogen, mixed with lysis buffer, and oscillated with a homogenizer at 4 °C. Oscillation was conducted once every 5 min for six times, and then centrifuged for 10 min. The supernatant was collected to extract proteins, and the protein concentration was determined using the BCA method. Loading buffer was added, and the mixture was boiled for 10 min for denaturation. Twenty micrograms of protein were loaded, subjected to Sodium Dodecyl Sulfate Polyacrylamide gel electrophoresis, and electrotransferred to a polyvinylidene fluoride membrane. The membrane was blocked with 50 g/L skim milk solution at room temperature for 3 h, incubated with primary antibody at 4 °C overnight, washed, and incubated with horseradish peroxidase-conjugated secondary antibody at room temperature for 1 h. After washing, the protein bands were visualized using electrochemiluminescence. GAPDH was used as an internal reference, and Image J analysis software was used to analyze the gray values of the bands in each group.

### 2.4 Data analysis

SPSS 26.0 statistical software (IBM, Armonk, NY, USA) was used for statistical analysis of the data in this study. The Shapiro-Wilk test was used to determine whether the data conformed to a normal distribution (*P* > 0.05 indicates a normal distribution). One-way analysis of variance was used to compare multiple groups with a normal distribution and homogeneous variance, the Kruskal-Wallis H test was used for data that did not meet the assumptions of normal distribution and variance homogeneity, and effect sizes (r) were calculated. All data were expressed as mean ± standard deviation. The results were considered statistically significant at *P* < 0.05. Graphs were constructed using GraphPad Prism 6.0. All experiments were independently replicated three times to ensure the accuracy of the experimental results.

## 3 Results

### 3.1 Network pharmacology

#### 3.1.1 Active components and potential targets of Heidihuang Pill

ADME-based screening produced 30 qualifying HP constituents: rehmanniae (two components), atractylodes (nine components), dried ginger (six components), and jujubae (19 components) (**Table 1**). Target proteins were annotated using UniProt, and 228 unique targets remained after deduplication.

**Table 1:** Main active ingredients of HP.

Mol. ID	Molecule name	Oral bioavailability (OB [%])	Drug- likeness (DL)	Drug
MOL000359	sitosterol	36.91	0.75	Rehmannia, Dried ginger
MOL000449	Stigmasterol	43.83	0.76	Rehmannia, Jujube
MOL000188	3β-acetoxyatractylone	40.57	0.22	Atractylodes
MOL000184	NSC63551	39.25	0.76	Atractylodes
MOL000173	wogonin	30.68	0.23	Atractylodes
MOL000085	beta-daucosterol_qt	36.91	0.75	Atractylodes
MOL007004	Albiflorin	30.25	0.77	Atractylodes
MOL001924	Paeoniflorin	53.87	0.79	Atractylodes
MOL000492	(+)-Catechin	54.83	0.24	Atractylodes, Jujube
MOL000358	beta-sitosterol	36.91	0.75	Atractylodes, Dried ginger, Jujube
MOL002813	Aucubin	35.56	0.33	Atractylodes
MOL002464	1-Monolinolein	37.18	0.3	Dried ginger
MOL002501	[(1S)-3-[(E)-but-2-enyl]-2-methyl-4-oxo-1-cyclopent-2-enyl] (1R,3R)-3-[(E)-3-methoxy-2-methyl-3-oxoprop-1-enyl]-2,2-dimethylcyclopropane-1-carboxylate	62.52	0.31	Dried ginger
MOL002514	Sexangularetin	62.86	0.3	Dried ginger
MOL000098	quercetin	46.43	0.28	Dried ginger, Jujube
MOL012921	stepharine	31.55	0.33	Jujube
MOL012946	zizyphus saponin I_qt	32.69	0.62	Jujube
MOL012976	coumestrol	32.49	0.34	Jujube
MOL012981	Daechuine S7	44.82	0.83	Jujube
MOL012986	Jujubasaponin V_qt	36.99	0.63	Jujube
MOL012992	Mauritine D	89.13	0.45	Jujube
MOL001454	berberine	36.86	0.78	Jujube
MOL001522	(S)-Coclaurine	42.35	0.24	Jujube
MOL000211	Mairin	55.38	0.78	Jujube
MOL004350	Ruvoside_qt	36.12	0.76	Jujube
MOL000627	Stepholidine	33.11	0.54	Jujube
MOL007213	Nuciferin	34.43	0.4	Jujube
MOL000787	Fumarine	59.26	0.83	Jujube
MOL002773	beta-carotene	37.18	0.58	Jujube
MOL000096	(-)-catechin	49.68	0.24	Jujube

#### 3.1.2 Osteoporosis-related target proteins

A Search for “Osteoporosis” identified 5,936 targets in GeneCards, 45 in OMIM, and 115 in the Drug Bank. These datasets were consolidated, standardized using UniProt, and deduplicated, resulting in 6,032 non-redundant targets for further analysis.

#### 3.1.3 Intersection targets and network construction

**Figure 1:**
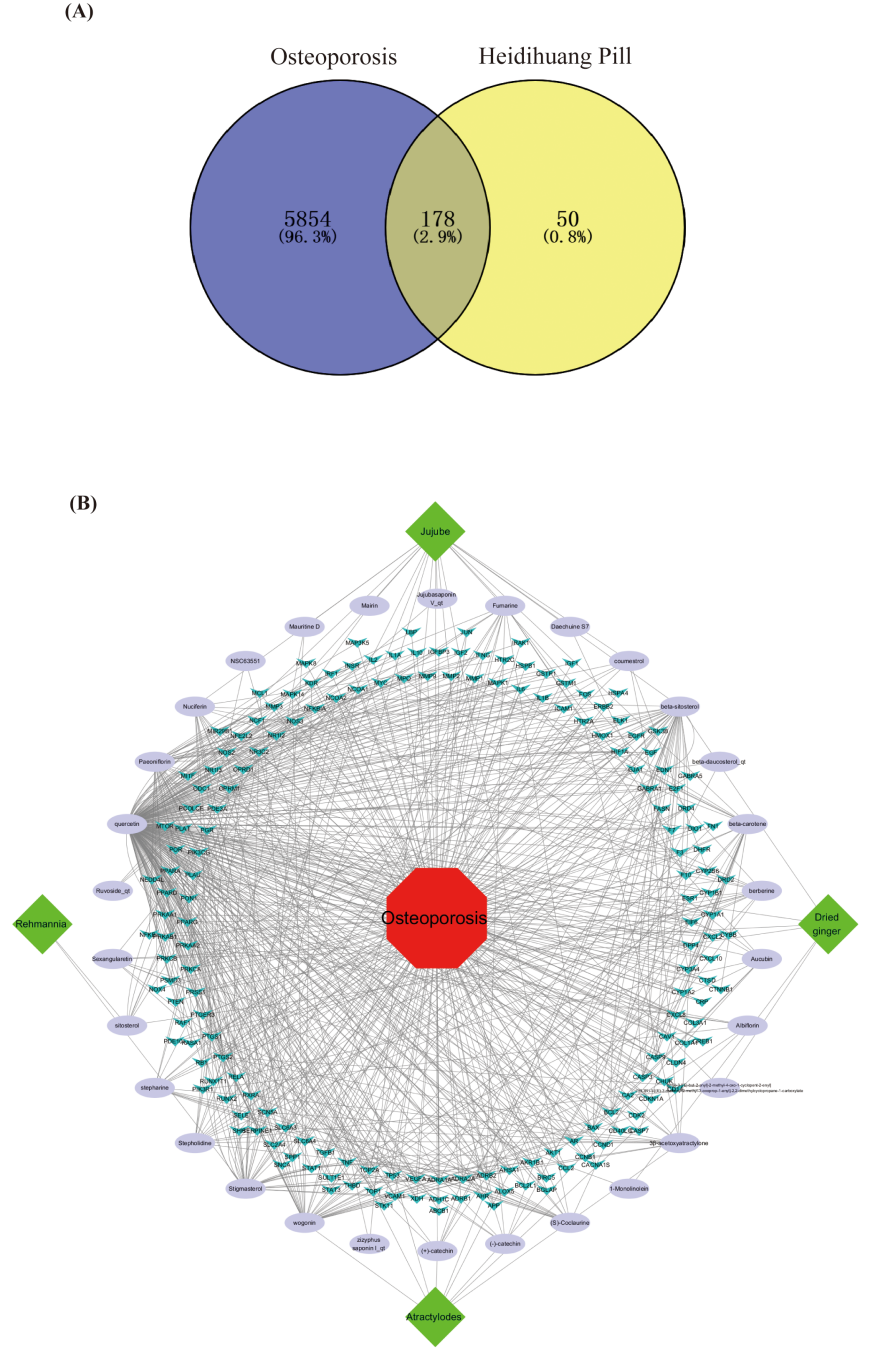
Intersection targets and network construction. **(A)** A Venn diagram formed by the mapping target proteins of Heidihuang Pill (HP) to the target proteins of OP. Overall, 50 target proteins associated with HP were identified, as well as 5854 target proteins related to Osteoporosis (OP). Overall, 178 intersecting target proteins between the two were obtained, which are considered the key targets for HP in the treatment of OP. **(B)** Drug-active ingredient-key target-disease network diagram. The network diagram contains 213 nodes and 601 edges, where green represents the drug composition in HP, purple nodes represent the effective active ingredients of HP, blue nodes represent key targets, red nodes represent OP, and edges represent the interactions between nodes.

Venny 2.1 identified 178 shared HP-OP targets (**Figure 1A**), which were designated as key therapeutic targets. A drug-ingredient-target-disease network was constructed in Cytoscape 3.7.1 (**Figure 1B**) using these targets, HP drugs, bioactive ingredients, and OP nodes. The network (213 nodes, 601 edges) used color coding: green (HP drugs), purple (bioactives), blue (targets), and red (OP), with edges indicating functional interactions. The diagram shows that the effective ingredients of HP intervene in OP by acting on key targets.

#### 3.1.4 Protein-protein interaction network analysis and core target screening

**Figure 2:**
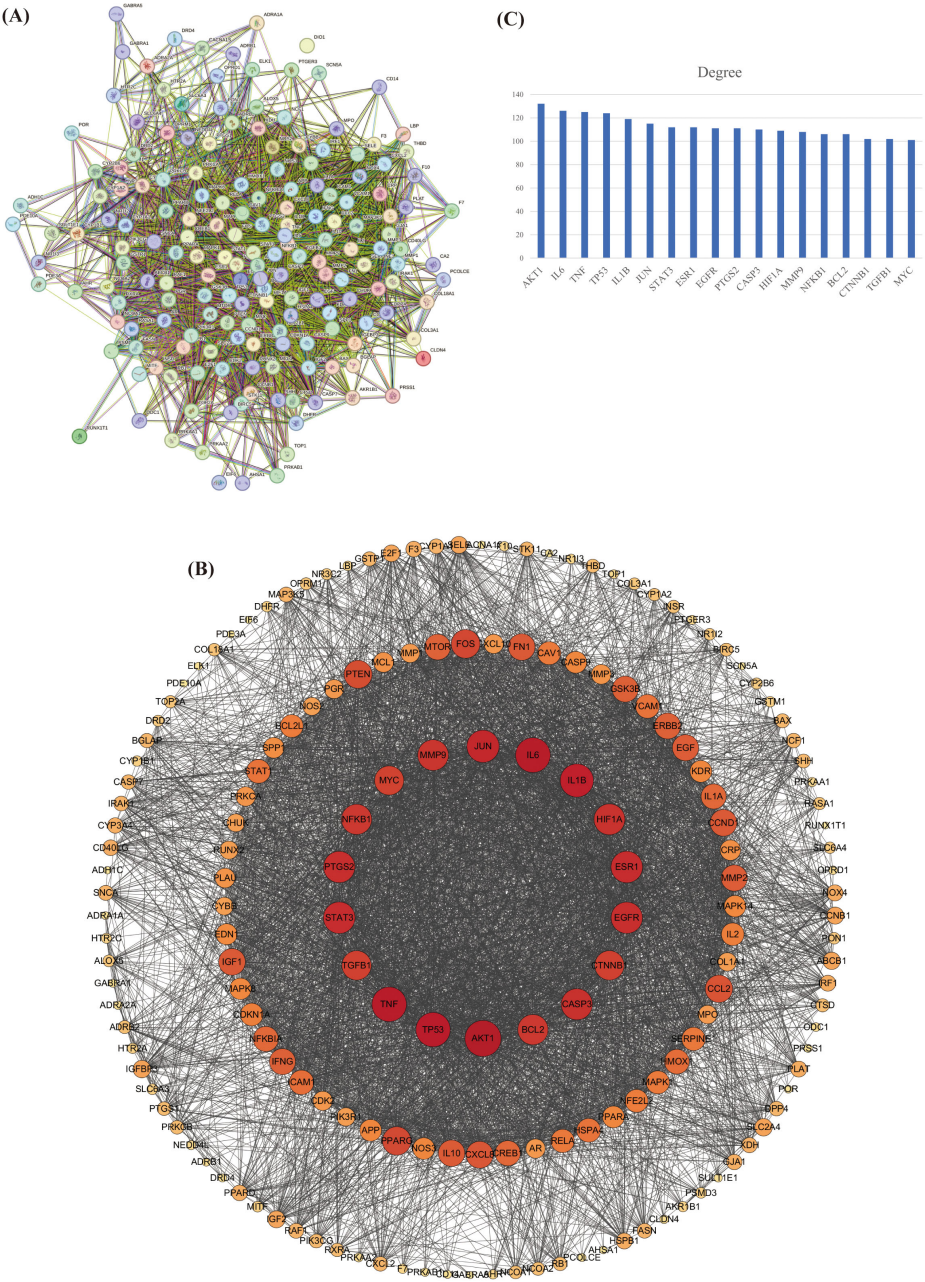
PPI network analysis and core target screening. **(A)** From the STRING platform, the Protein-Protein Intervention network was constructed, which included 177 nodes and 4281 edges. **(B)** The results of the STRING platform were imported into the network diagram obtained by Cytoscape 3.7.1 software. Node color depth and size reflect target importance. **(C)** Bar graphs were created based on the top 20 target proteins ranked by the degree value. Akt1 ranked at the top.

The overlapping targets of HP and OP were imported into the STRING platform to create a PPI network (**Figure 2A**). This network included 177 nodes and 4281 edges. Cytoscape 3.7.1 topological analysis prioritized biologically significant hub genes using the degree of centrality (DC) calculated using CytoNCA. A filtered subnetwork (**Figure 2B**) visualizes the target importance through graduated node sizes and colors. A bar chart was created following the top 20 target proteins ranked by DC (**Figure 2C**). As shown in the bar chart, RAC-alpha serine/threonine-protein kinase (Akt1) ranks among the top targets. Therefore, Akt1 is a key target of HP for treating OP.

#### 3.1.5 Biological functions and major signaling pathways of common targets

**Figure 3:**
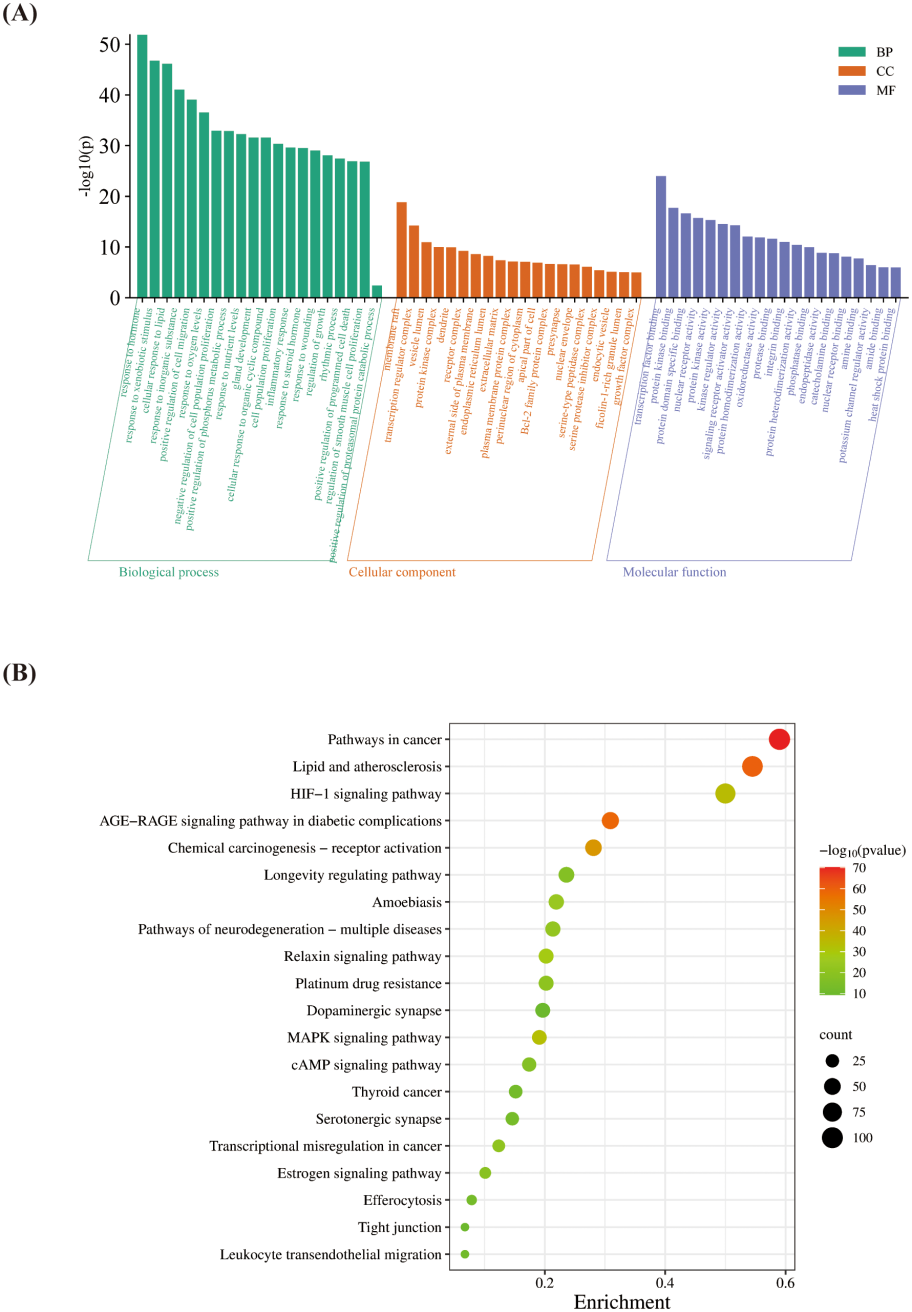
GO functional enrichment analysis and KEGG pathway enrichment analysis. **(A)** Based on gene ontology (GO) functional enrichment analysis, the top 20 items were selected for analysis to generate a bar graph. The x-axis represents three GO terms: Biological processes, cellular components, and molecular functions. The y-axis represents −log10 (p-value) of GO terms. The −log (p-value) of GO terms was indicated numerically above the bar plot. **(B)** Kyoto Encyclopedia Gene and Genome pathway enrichment analysis was conducted, and a bubble graph was generated. The number of genes enriched in each pathway was represented by the circle size, from green to red, representing the change in p-value.

The intersecting targets of HP and OP were uploaded to the Metascape platform for GO functional enrichment analysis; the top 20 items were selected for analysis, and a bar chart was drawn (**Figure 3A**). Using the screening criteria of Min Overlap: 3, P-Value Cutoff: 0.01, and Min Enrichment: 1.5, KEGG pathway enrichment analysis was conducted, the top 20 representative pathways were selected as key pathways, and they were visualized by drawing a bubble chart (**Figure 3B**). GO functional analysis revealed that HP regulates OP mainly by responding to hormones in biological processes, acting on membrane rafts in cellular components, and binding to transcription factors in molecular functions. KEGG pathway enrichment analysis of the intersecting targets revealed that the primary pathways involved included pathways in cancer, lipid, and atherosclerosis, the Advanced Glycation End products/Receptor for Advanced Glycation End products (AGE-RAGE) signaling pathway in diabetic complications, chemical carcinogenesis-receptor activation, and the HIF-1 signaling pathway.

### 3.2 Molecular docking

**Figure 4:**
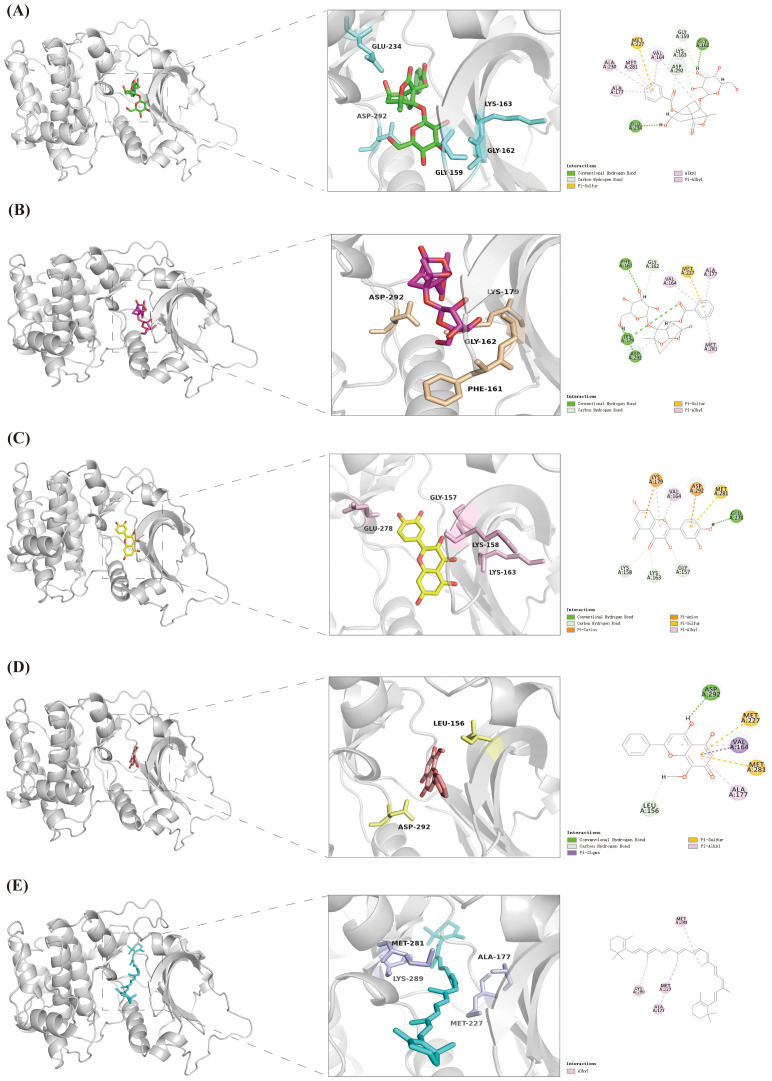
Molecular docking. **(A)** Albiflorin with Akt1, **(B)** Paeoniflorin with Akt1, **(C)** Quercetin with Akt1, **(D)** Wogonin with Akt1, **(E)** Beta-Carotene with Akt1.

Previous studies indicated that Akt1 (PDB ID: 4GV1) is the main target. Five active components of HP — Albiflorin [PubChem CID: 24868421], Peoniflorin [PubChem CID: 442534], Quercetin [PubChem CID: 5280343], Wogonin [PubChem CID: 5281703], and Beta-Carotene [PubChem CID: 5280489] — were selected for molecular docking analysis. The results showed that the binding energies of Akt1 with Albiflorin, Peoniflorin, Quercetin, Wogonin, and Beta-Carotene were -56.20 kcal/mol, -56.66 kcal/mol, -41.93 kcal/mol, -35.83 kcal/mol, and -6.97 kcal/mol, respectively. It is generally believed that the higher the absolute value of the binding energy, the stronger the affinity between the two (22). These interactions are mediated by forces, including conventional hydrogen bonds, carbon-hydrogen bonds, alkyl bonds, and Pi-alkyl bonds. Before formal docking, all macromolecular structures were redocked, and their root mean square deviation values were all <2 Å, indicating good accuracy and a favorable binding mode (23). (**Figure 4** and **Table 2**).

**Table 2:** Molecular docking of AKT1 and HP active ingredients.

HP active ingredients	Connection method and location	Binding energy (kcal/mol)	Root mean square deviation(Å)
Albiflorin	Conventional Hydrogen Bond (CLY (A:162), GLU (A:234)); Carbon Hydrogen Bond (GLY(A:159), LYS(A:163), ASP(A:292)); Pi-Sulfur (MET(A:227)); Alkyl, Pi-Alkyl (ALA (A:177, A:230), MET (A:281), VAL (A:164))	-56.20	1.14
Peoniflorin	Conventional Hydrogen Bond (PHE (A:161), LYS (A:179), ASP (A:292)); Carbon Hydrogen Bond (GLY (A:162); Pi-Sulfur (MET (A:227)); Pi-Alkyl (ALA (A:177), MET (A:281), VAL (A:164))	-56.66	0.97
Quercetin	Conventional Hydrogen Bond (GLU(A:278)); Carbon Hydrogen Bond (GLY (A:157), LYS (A:163, A:158)); Pi-Cation, Pi-Anion (LYS (A:179), ASP (A:292)); Pi-Sulfur (MET (A:281)); Pi-Alkyl (VAL (A:164))	-41.93	1.53
Wogonin	Conventional Hydrogen Bond (ASP (A:292)); Carbon Hydrogen Bond (LEU (A:156)); Pi-Sigma (VAL (A:164)); Pi-Sulfur (MET (A:281, A:227)); Pi-Alkyl (VAL (A:164))	-35.83	1.56
Beta-Caroten	Alkyl (LYS (A:289), MET (A:281, A:227), ALA (A:177))	-6.97	1.64

### 3.3 Experimental validation

#### 3.3.1 Micro-computed tomography analysis of femoral bone in rats

**Figure 5:**
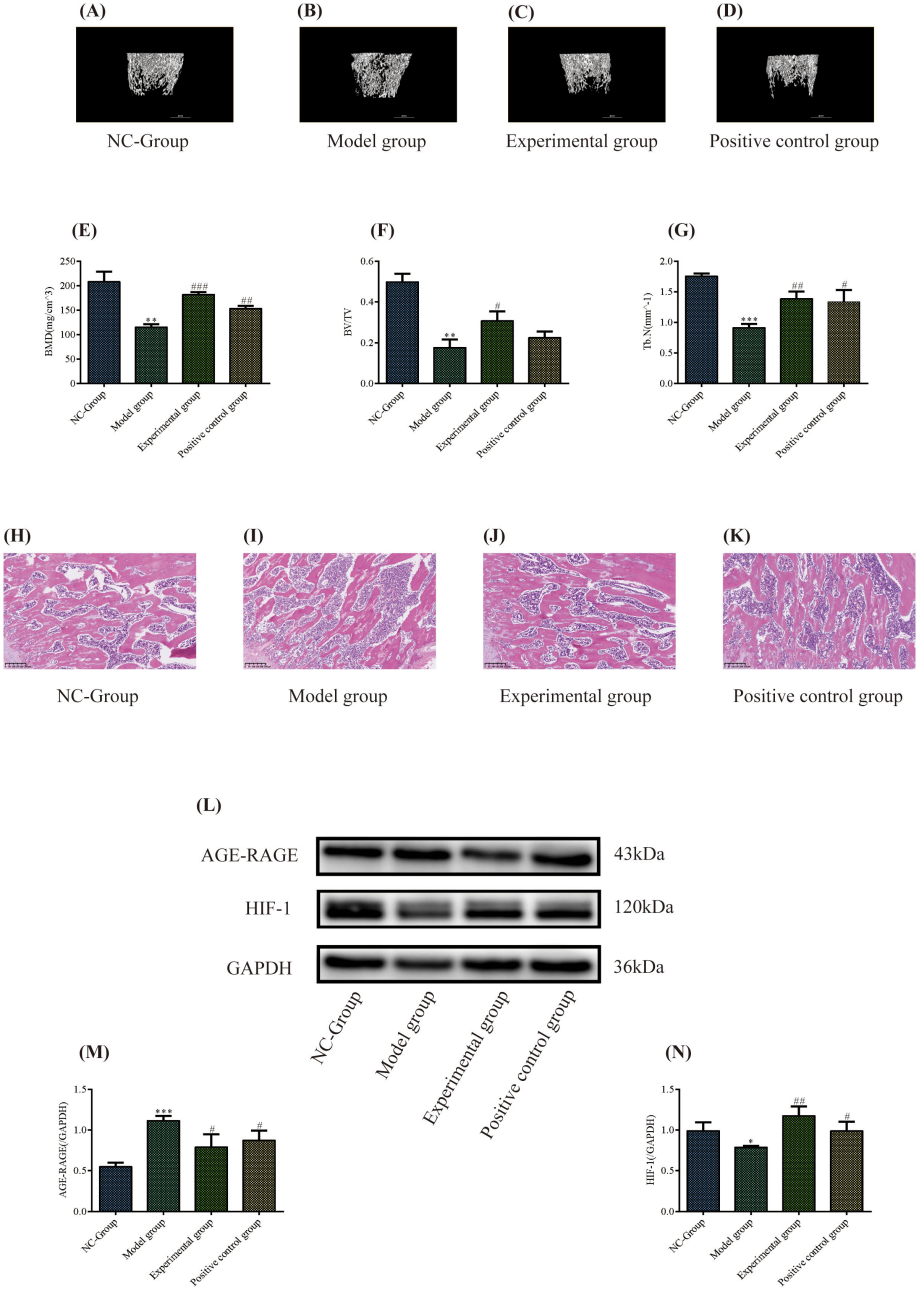
Experimental validation. **(A–D)** Three-dimensional reconstruction of the distal femur by Micro-Computed Tomography. **(E–G)** Quantitative analysis of bone microstructure (BMD, BV/TV, Tb.N). *P < 0.05, **P < 0.01, **P < 0.001 versus the NC-group; #*P* < 0.05, ##*P* < 0.01, ###*P* < 0.001 versus the model group. **(H–K)** Histological Evaluation of Femoral Bone via Hematoxylin and Eosin Staining. **(L–N)** Western blotting was used to detect the expression of the signaling proteins Advanced Glycation End Products/Receptor for Advanced Glycation End Products and HIF-1. Protein concentration analysis. *P < 0.05, **P < 0.01, ***P < 0.001 versus the NC-group; #*P* < 0.05, ##*P* < 0.01, versus the model group.

Three-dimensional reconstruction of the distal femur using micro-CT revealed that compared to the NC-Group, the model group showed significant reductions in trabecular bone quantity, disrupted continuity, and enlarged trabecular spacing. Conversely, the experimental and positive control groups showed increased trabecular bone density, improved trabecular continuity, and reduced trabecular spacing than the model group (**Figures 5A–D**).

Quantitative analysis of the bone microstructure showed that the model group had significantly lower trabecular BMD, Tb.N, and BV/TV than the NC group did (*P* = 0.002, 0.000, 0.002 < 0.05, r=0.95, 0.99, 0.97, respectively). Compared to the model group, the BMD, Tb.N, and BV/TV of rats in the experimental group were significantly higher (*P* = 0.000, 0.004, 0.021< 0.05, r=0.99, 0.93, 0.83, respectively). In the positive control group, the BMD and Tb.N of rats showed an upward trend (*P* = 0.001, 0.022 < 0.05, r=0.95, 0.83, respectively), while the upward trend of BV/TV was not statistically significant (*P* = 0.170 > 0.05, r= 0.56) (**Figures 5E–G**).

#### 3.3.2 Histological evaluation of femoral bone via hematoxylin and eosin staining

Compared with the control group, the model group showed significantly reduced trabecular bone quantity, thinned trabecular morphology, widened and fragmented intertrabecular spaces, and disrupted trabecular networks. Conversely, the experimental and positive control groups showed increased trabecular bone quantity, thickened trabecular morphology, enhanced continuity, and partial restoration of the trabecular network compared with the model group. Notably, the experimental group showed markedly thickened trabecular morphology, while the positive control group showed improved trabecular quantity and more pronounced network restoration (**Figures 5H–K**).

#### 3.3.3 Heidihuang Pill affects the expression of advanced glycation end products/receptor for advanced glycation end products signaling pathway and hypoxia-inducible factor-1 signaling pathway proteins in bone tissue

The expression of the AGE-RAGE signaling pathway and HIF-1 signaling pathway proteins in bone tissue were examined to further validate the results of the network pharmacology KEGG analysis and investigate the potential mechanism by which HP improves OP. The results showed (**Figures 5L–N**) that, compared to the NC-Group, the expression of HIF-1 signaling pathway proteins in the model group was significantly decreased (*P* = 0.028 < 0.05, r=0.81), while the expression of AGE-RAGE signaling pathway proteins significantly increased (*P* = 0.000 < 0.05, r=0.98). Compared with the model group, the expression of HIF-1 signaling pathway proteins in the experimental group increased (*P* = 0.005 < 0.05, r=0.92), and the expression of AGE-RAGE signaling pathway proteins was significantly decreased (*P* = 0.028 < 0.05, r=0.81); in the positive control group, the expression of HIF-1 signaling pathway proteins increased (*P* = 0.036 < 0.05, r=0.98), and the expression of AGE-RAGE signaling pathway proteins was significantly decreased (*P* = 0.033 < 0.05, r=0.79). The experimental group showed a more significant effect on upregulating the proteins of the HIF-1 signaling pathway and a more obvious effect on downregulating the expression of AGE-RAGE signaling pathway proteins. These results indicate that HP can activate the HIF-1 signaling pathway and inhibit the AGE-RAGE signaling pathway, which may be a potential mechanism for treating OP with HP.

## 4 Discussion

The incidence of OP increases with age, making it a significant public health challenge (24). In clinical practice, the long-term use of Western medications for OP has been associated with an increased incidence of adverse effects over time (25), and abrupt discontinuation of these drugs can lead to a sharp decline in bone mineral density (26). Over thousands of years of clinical validation, TCM has shown remarkable effects in treating OP, characterized by high therapeutic effectiveness, minimal side effects, and reduced recurrence rates (27, 28). HP, first documented in the ancient medical text Su Wen (The Yellow Emperor’s Classic of Medicine), was used for centuries as a formula to strengthen the spleen and nourish the kidneys. It is also a potent remedy for improving OP. However, the specific mechanisms underlying the therapeutic effects of HP in OP remain unclear. Therefore, a comprehensive investigation of the HP’s pharmacological mechanisms in treating OP, integrating network pharmacology, molecular docking, and experimental validation, is important for advancing OP management and therapeutic outcomes.

Network pharmacology was used to study the targets and mechanisms of action of HP in OP treatment. The results showed that the 30 active components of HP exerted therapeutic effects on OP by acting on 178 targets. PPI analysis was conducted on these 178 target proteins, which were ranked based on their DC. Among these, the degree value of the Akt1 ranked first; therefore, we believe that Akt1 is the core target of HP for treating OP. Akt1, also known as protein kinase B, is an important node in many signaling pathways and regulates various biological and pathological processes such as cell proliferation and energy metabolism (29). Previous studies have shown that induced Akt1 expression promotes the proliferation of mesenchymal stem cells and ultimately inhibits their apoptosis, thereby alleviating osteoporosis (30). Additionally, studies have reported that Akt1 is essential in the pathological process of OP: regulating Akt1 signaling can enhance the differentiation and function of osteoclasts and inhibit osteoclast formation by regulating key regulators of osteoclastogenesis, thus playing an important role in OP (31–33). Akt1 plays a key role in OP by participating in multiple signaling pathways.

Molecular docking is an effective method for showing interactions between small molecules and proteins. The interactions between the candidate active ingredients and key targets were further verified through molecular docking simulations, providing sufficient evidence for treating OP with HP. Molecular docking was conducted between the target Akt1 and five related active ingredients, and peoniflorin was found to have a stronger binding affinity with Akt1. Therefore, Peoniflorin is considered the main active ingredient of HP for treating OP. Peoniflorin is a monoterpene glycoside extracted from the Chinese herbal medicine Paeonia lactiflora. It shows various pharmacological effects, including antioxidant, immunomodulatory, anti-inflammatory, anticancer, antidepressant-like, and neuroprotective effects (34). Studies have shown that Peoniflorin reduces osteoclast formation by regulating the NF-κB signal and stimulates osteoblast formation through the Wnt/β-catenin pathway, indicating its potential bone-protective effect in osteolytic diseases (35, 36). Peoniflorin promotes AKT1 expression, which further exerts a therapeutic effect.

KEGG pathway enrichment analysis revealed that 178 target proteins were enriched in 20 pathways. Among these, the AGE-RAGE and HIF-1 signaling pathways ranked among the top and were associated with OP. Animal experiments were conducted to verify the mechanism of action of HP in OP treatment. The results showed that the number of trabecular bones in the osteoporotic rat model increased significantly, the continuity of trabecular bones improved, and indicators such as trabecular bone mineral density, bone volume fraction, and trabecular number of the rat femur increased significantly after HP intervention. Finally, by detecting the expression of HIF-1 and AGE/RAGE proteins in the bone tissue, we discovered that the expression of HIF-1 signaling protein in the bone tissue of model rats after HP intervention increased. However, the expression of the AGE/RAGE signaling protein decreased. This shows that HP can improve OP by regulating the AGE-RAGE signaling pathway and the HIF-1 signaling pathway.

AGEs are a group of destructive compounds formed gradually under hyperglycemic conditions and are widely present in the extracellular matrix. AGEs are components of the extracellular matrix and are intracellular proteins. Their interactions with certain proteins can impair protein function. Additionally, AGEs to RAGE binding is a classical pathogenic pathway that triggers various diseases, including OP (37, 38). Elevated AGE levels suppress osteoblast proliferation and function by modulating autophagy via the AGE-RAGE pathway (39). Furthermore, increased AGE expression promotes osteoclastogenesis and impairs matrix mineralization (40), positioning AGEs as the “missing link” in explaining the heightened skeletal fragility associated with OP (41). Studies indicated that inhibiting AGE-RAGE signaling activation and mitigating its downstream effects can delay OP progression. For instance, Sun et al. showed that suppressing AGE-RAGE signaling alleviates bone loss and enhances bone microstructure by ameliorating bone marrow stromal cell dysfunction (42).

HIF-1 is a key transcriptional regulator of cellular responses to hypoxia that includes HIF-1α and HIF-1β subunits. It interacts with hypoxia-response elements to regulate the expression of target genes (43, 44). The HIF-1 signaling pathway, a complex regulatory network activated under hypoxic conditions, is critical for cell survival and adaptation to low-oxygen environments (45). It is important for energy metabolism and preventing skeletal metabolic disorders (43, 46). Bone homeostasis, a central factor in OP, relies on the balance between osteoblasts and osteoclasts, both of which are oxygen-sensing cells (47). The activation of HIF-1 signaling promotes osteoblast differentiation (48) and reduces osteoclast activity (49), thereby mitigating bone microstructural deterioration and bone loss. Bone tissue is highly vascularized, with blood vessels supplying nutrients and oxygen essential for osteoblast survival and activity, highlighting the importance of the vasculature in skeletal development (50). HIF-1 is a key regulator of H-type vessel formation. When HIF-1 accumulates to sufficient levels, it induces H-type vessel angiogenesis, which recruits osteoprogenitor cells and promotes bone regeneration through the coupling of angiogenesis and osteogenesis (51, 52). Recent studies have highlighted that activating the HIF-1 pathway enhances H-type vessel formation and improves OP outcomes (53).

In conclusion, this is the first study to investigate the efficacy and mechanism of action of HP in treating OP by combining network pharmacology, molecular docking, and experimental animal validation. The results revealed the potential of HP in treating OP, providing a scientific basis and new research direction for TCM intervention in OP. Our study has some limitations. Network pharmacology and molecular docking rely on data and algorithms; owing to the constraints of databases and software, their results may vary from the actual outcomes. Additionally, limited by time and resources, all the predicted targets, pathways, and downstream mechanisms of these pathways could not be experimentally confirmed. This prevented us from completely elucidating the anti-OP mechanism of the HP. Additional experiments will be conducted to examine the potential molecular pathways underlying the anti-OP effects of HP in greater detail.

## 5 Conclusion

This study is the first to systematically show that HP improves OP by acting on the Akt1 target through its main active component, paeoniflorin, and regulating the AGE-RAGE and HIF-1 signaling pathways, which deepens the pharmacological basis of HP in the treatment of OP from the perspective of multiple components, targets, and pathways, and also provides a mechanistic basis for the precise drug development of this compound. Future research should focus on improving the *in vivo* bioavailability of paeoniflorin, improving its dosage form, and conducting clinical verification of related signaling pathways to promote HP transformation into modern and standardized anti-OP drugs.

## Data Availability

The original contributions presented in the study are included in the article/supplementary material. Further inquiries can be directed to the corresponding author.
