# Supplementary figures and images for: Exploring the mechanism of Heidihuang Pill in the treatment of osteoporosis based on network pharmacology, molecular docking, and experimental validation
Source: Front Endocrinol (Lausanne). 2025 Dec 15;16:1664254. doi: 10.3389/fendo.2025.1664254 (PMC12745157; doi:10.3389/fendo.2025.1664254)

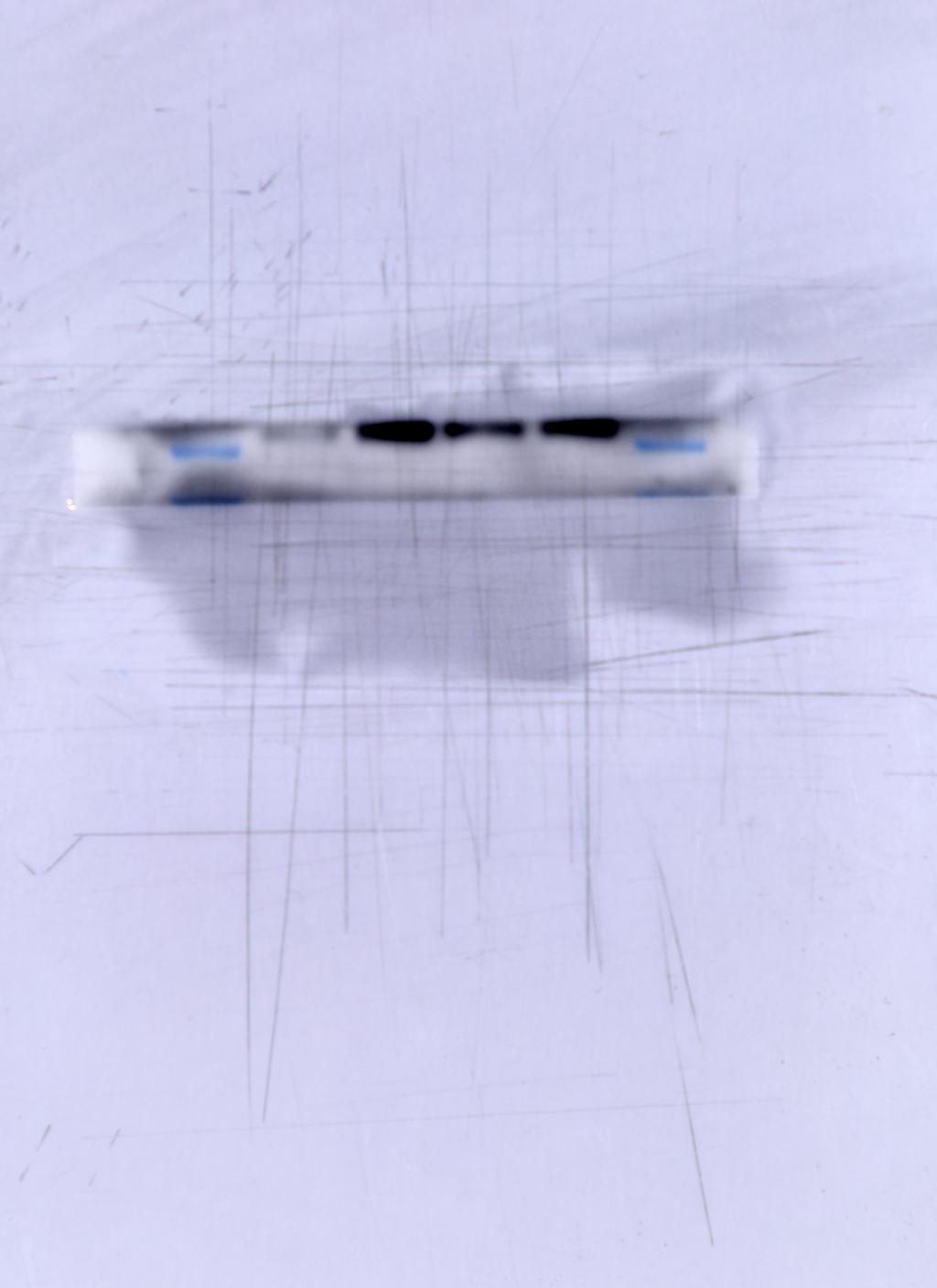

Supplement: Supplementary file 1 [file DataSheet1.zip › raw data/WB/age-rage 1 marker.jpg]

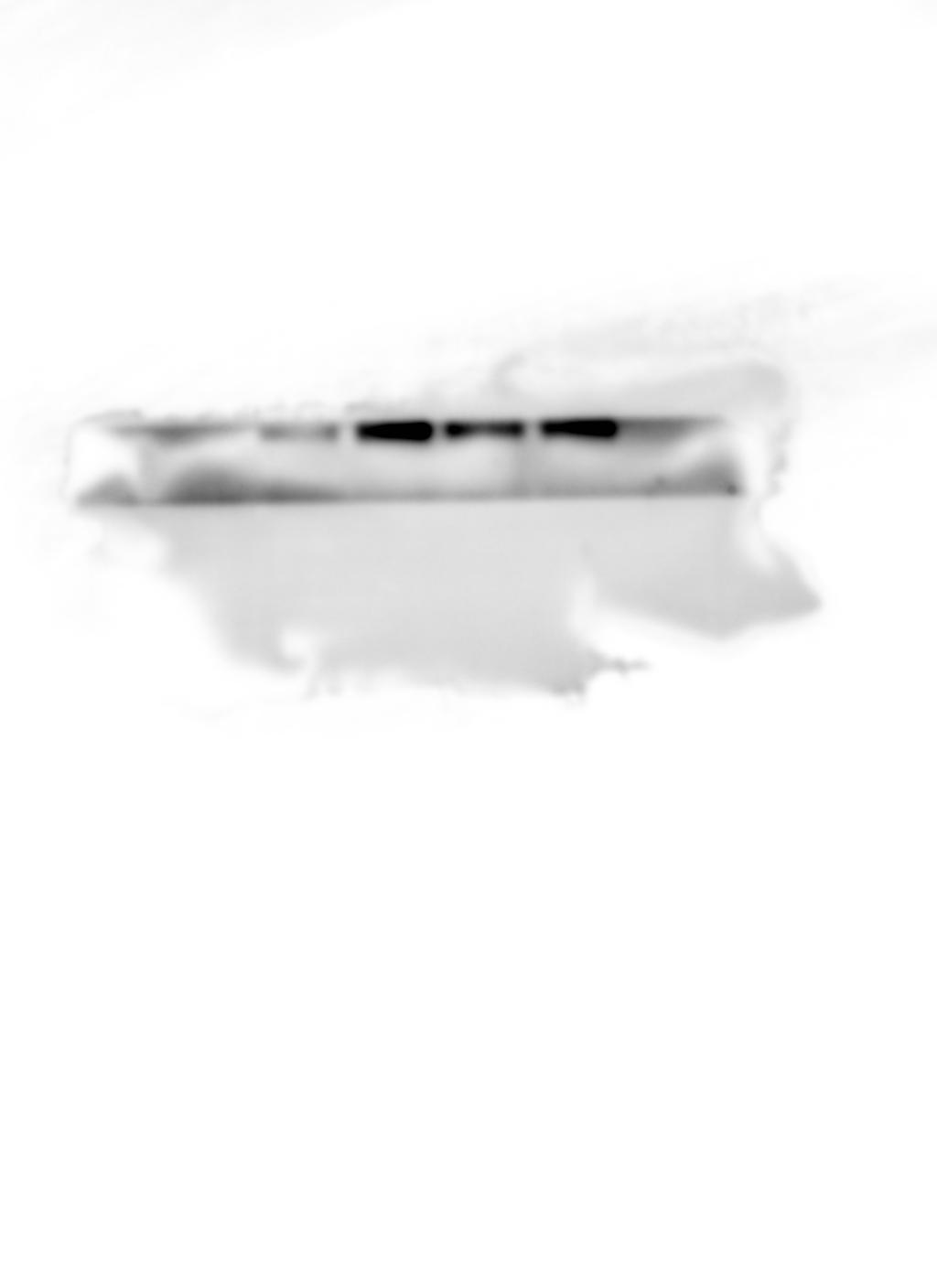

Supplement: Supplementary file 1 [file DataSheet1.zip › raw data/WB/age-rage 1.jpg]

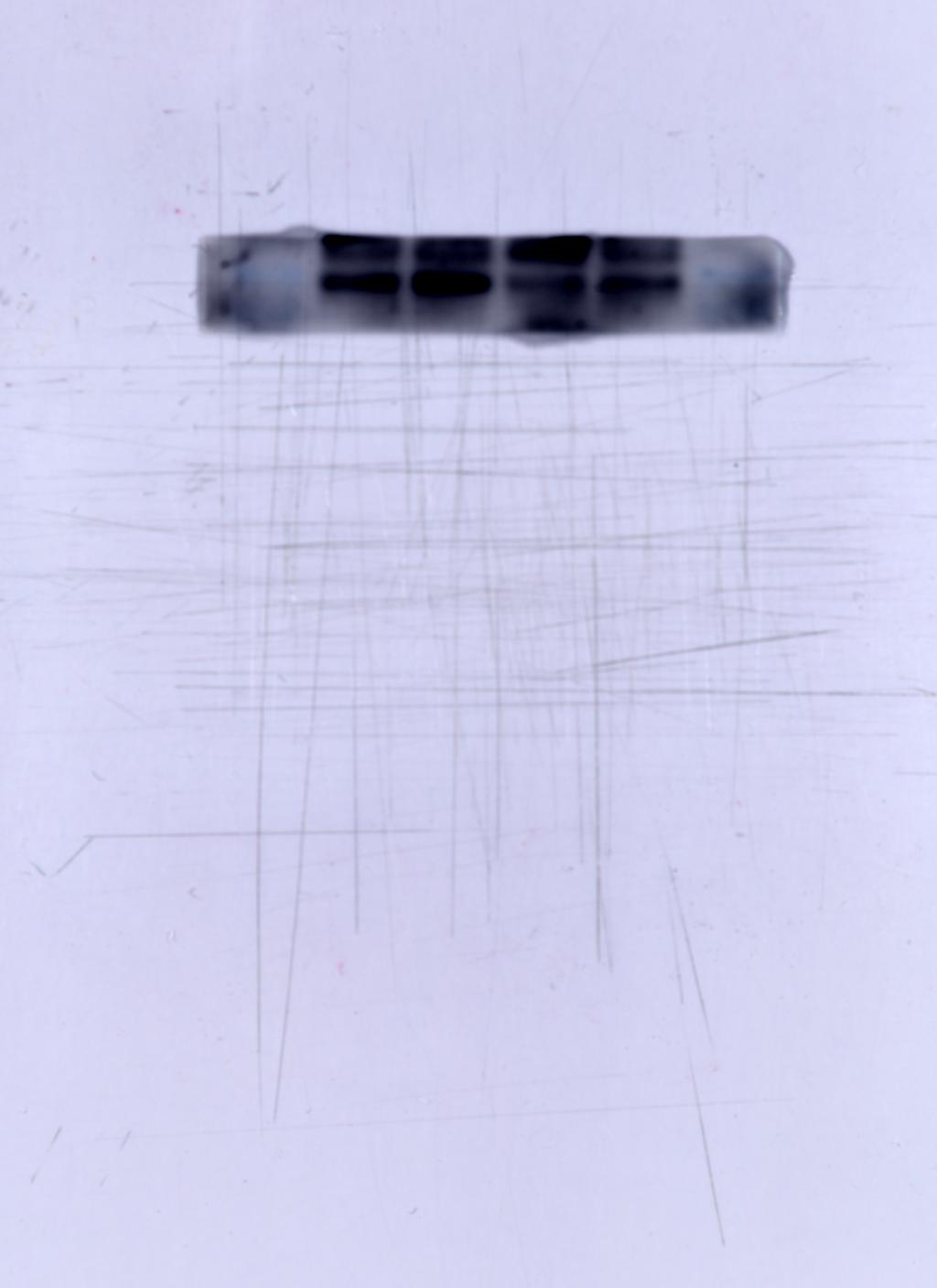

Supplement: Supplementary file 1 [file DataSheet1.zip › raw data/WB/age-rage2 marker.jpg]

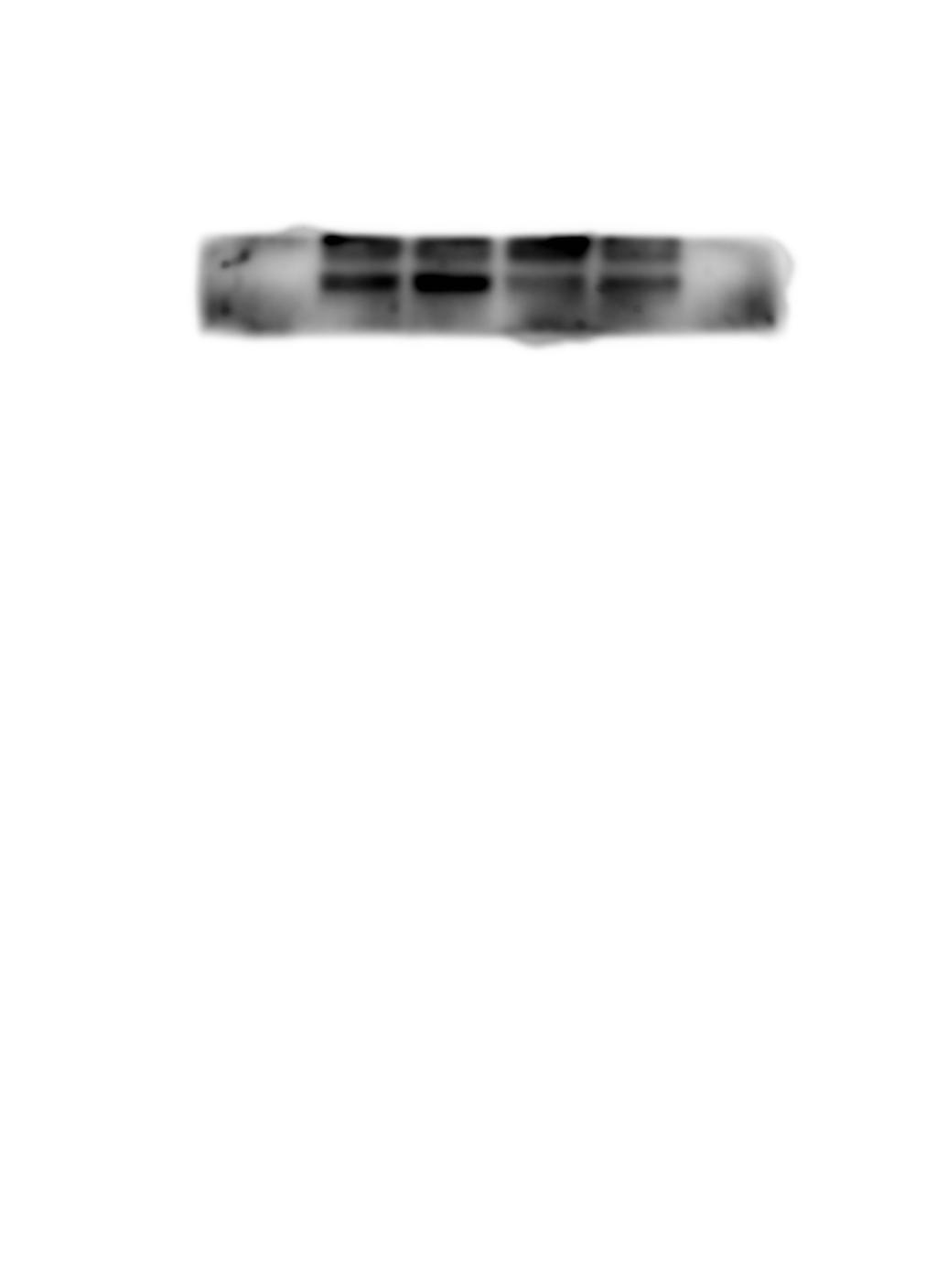

Supplement: Supplementary file 1 [file DataSheet1.zip › raw data/WB/age-rage2.jpg]

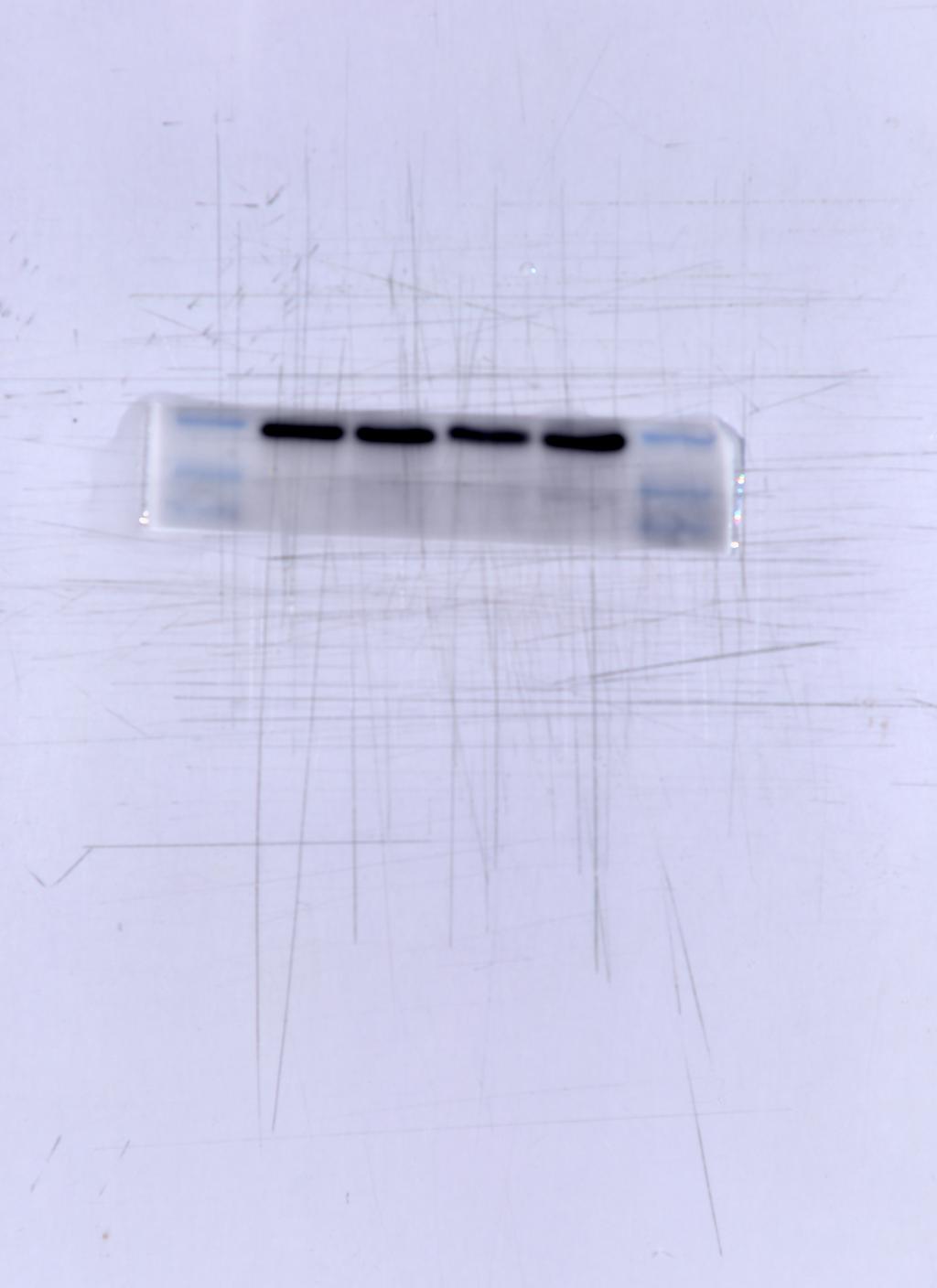

Supplement: Supplementary file 1 [file DataSheet1.zip › raw data/WB/age-rage3 marker.jpg]

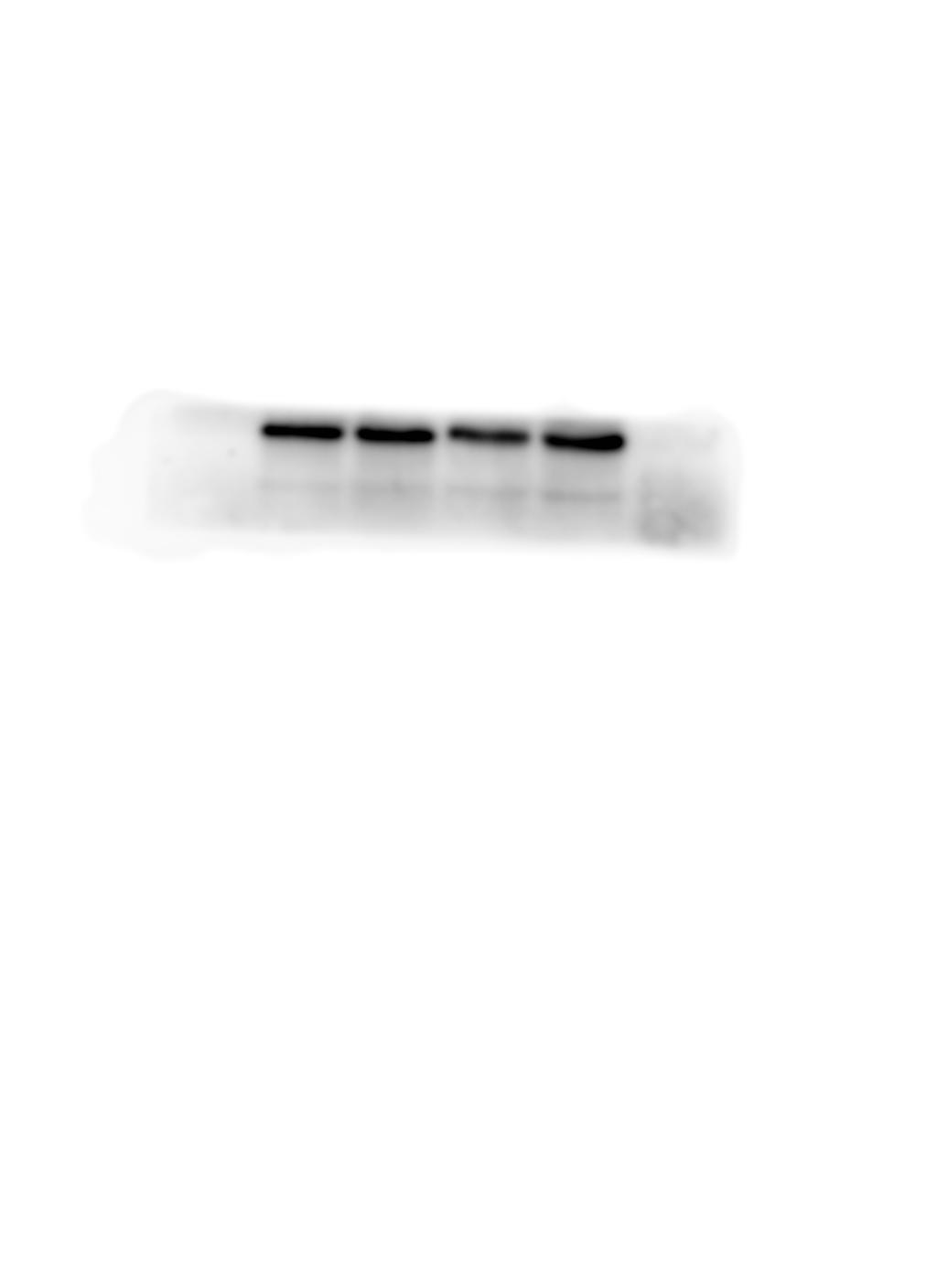

Supplement: Supplementary file 1 [file DataSheet1.zip › raw data/WB/age-rage3.jpg]

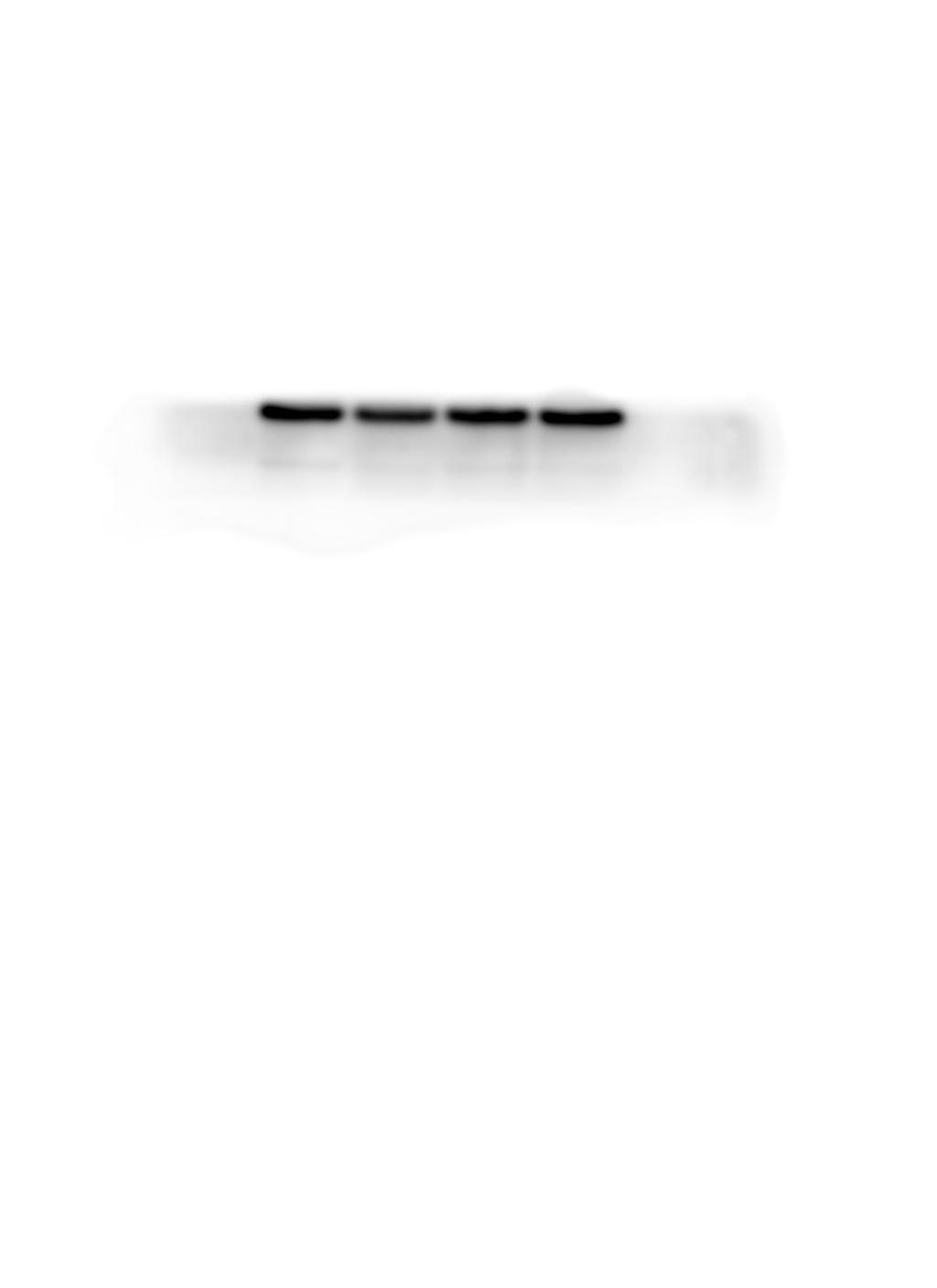

Supplement: Supplementary file 1 [file DataSheet1.zip › raw data/WB/GAPDH 1 .jpg]

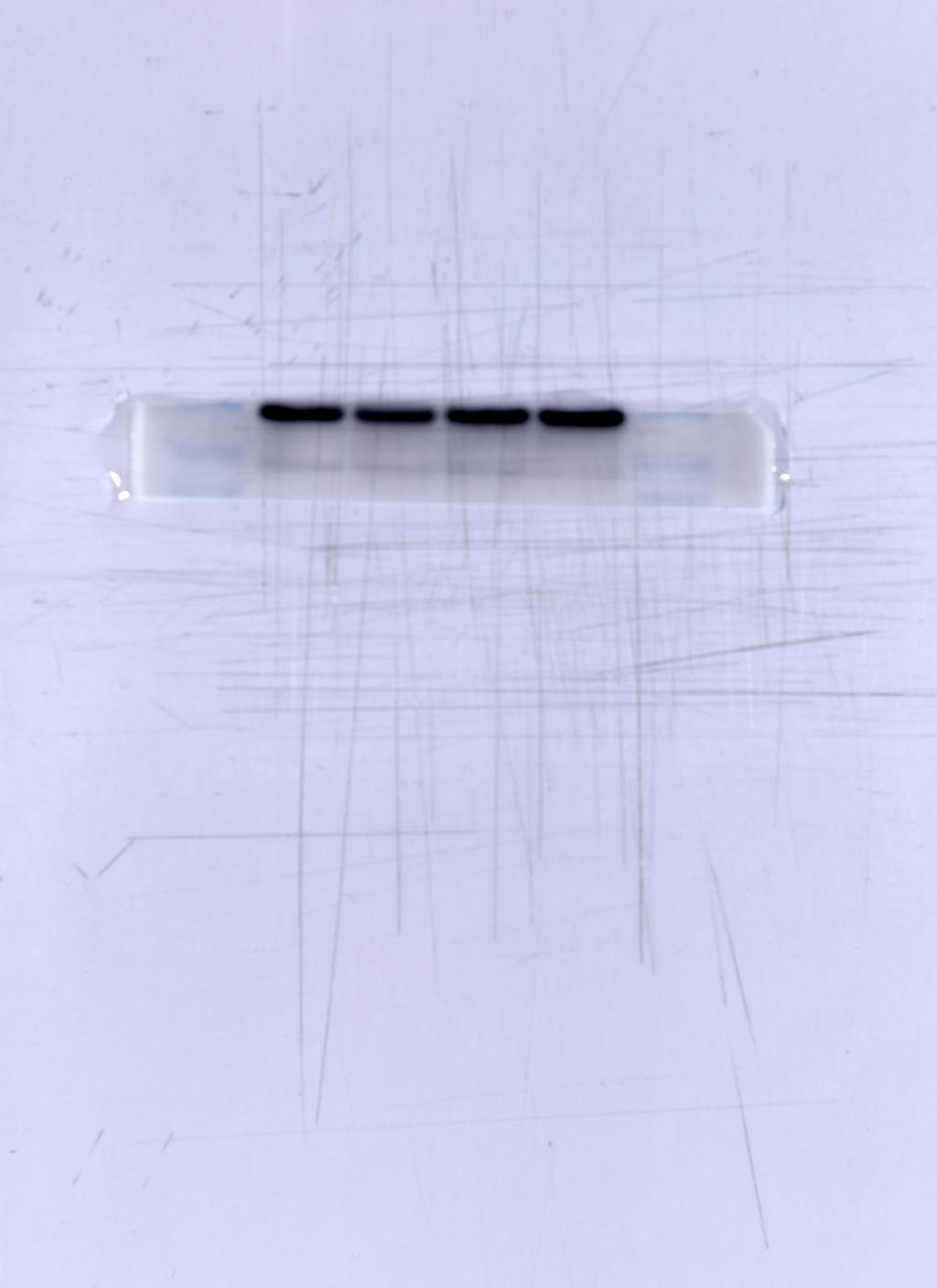

Supplement: Supplementary file 1 [file DataSheet1.zip › raw data/WB/GAPDH 1 Marker.jpg]

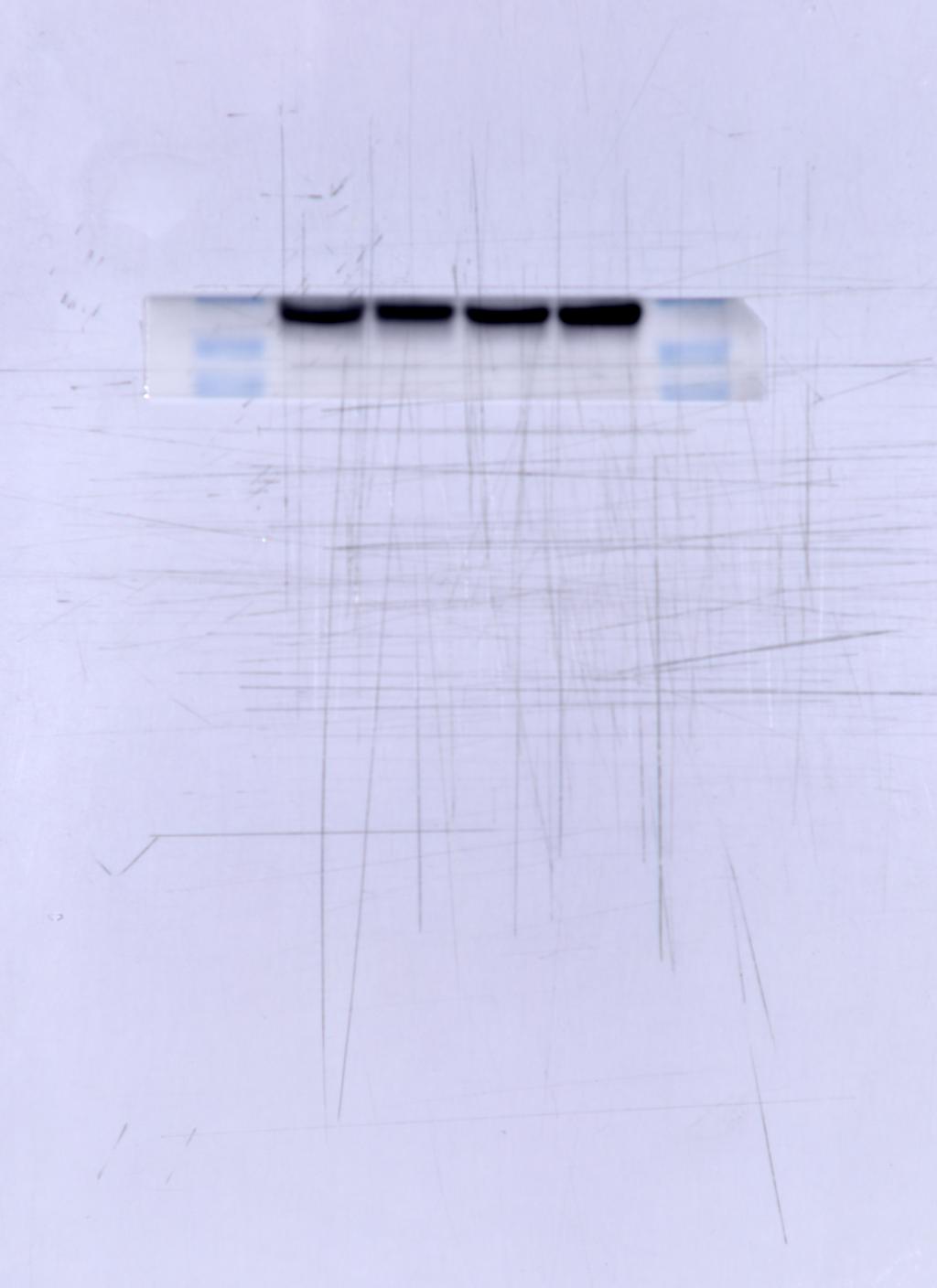

Supplement: Supplementary file 1 [file DataSheet1.zip › raw data/WB/GAPDH 2 Marker.jpg]

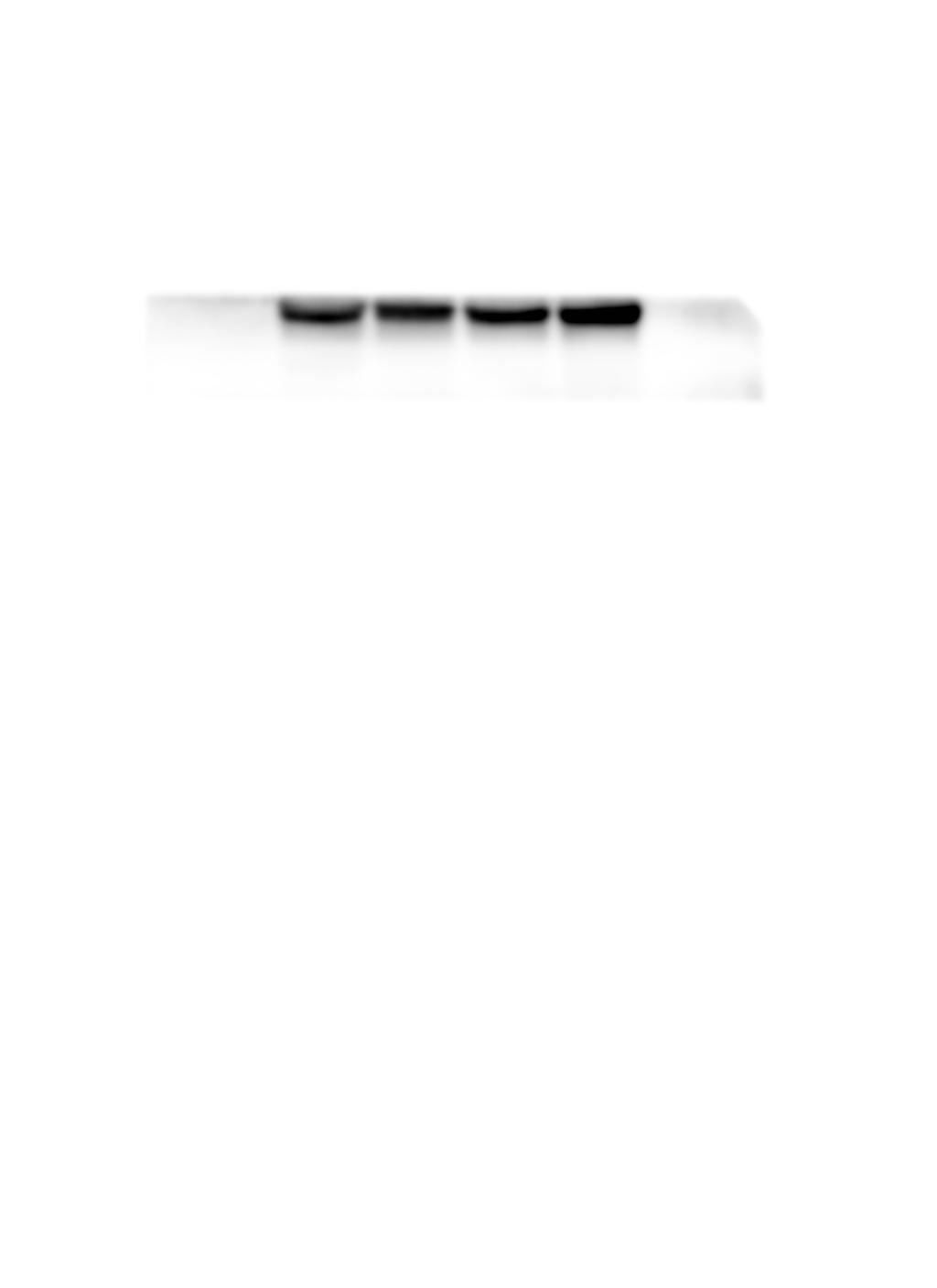

Supplement: Supplementary file 1 [file DataSheet1.zip › raw data/WB/GAPDH 2.jpg]

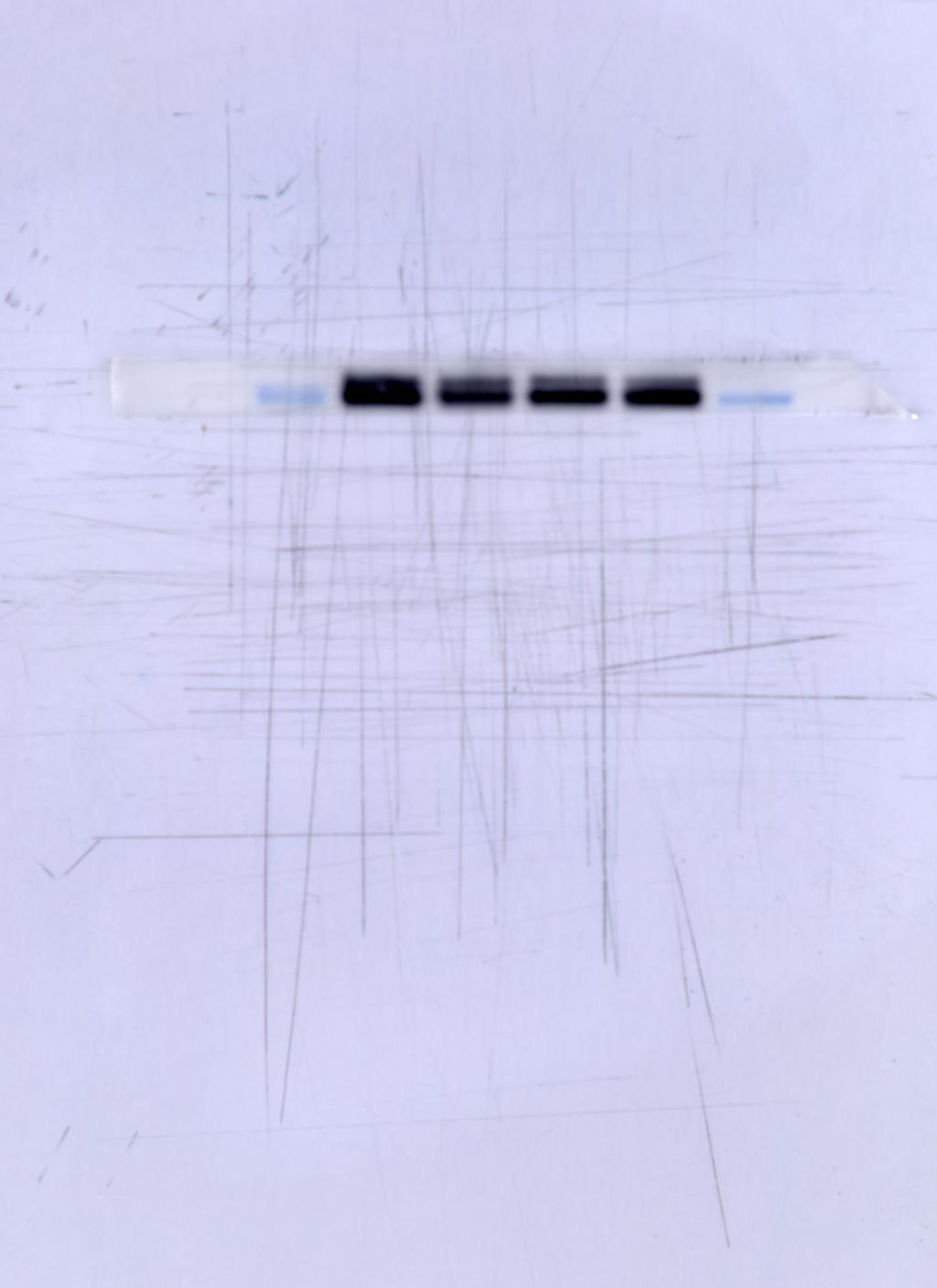

Supplement: Supplementary file 1 [file DataSheet1.zip › raw data/WB/hif-1 1 marker .jpg]

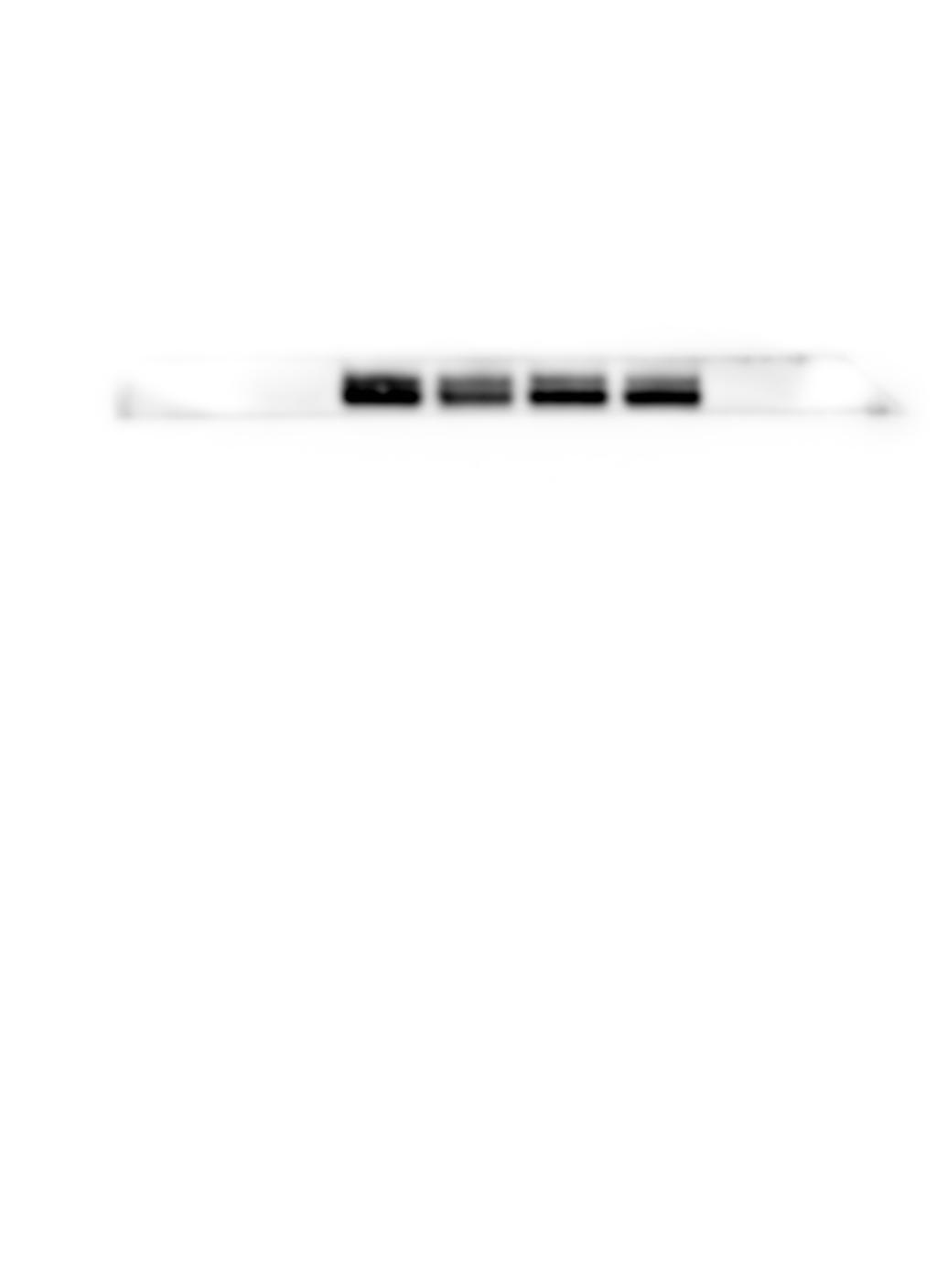

Supplement: Supplementary file 1 [file DataSheet1.zip › raw data/WB/hif-1 1.jpg]

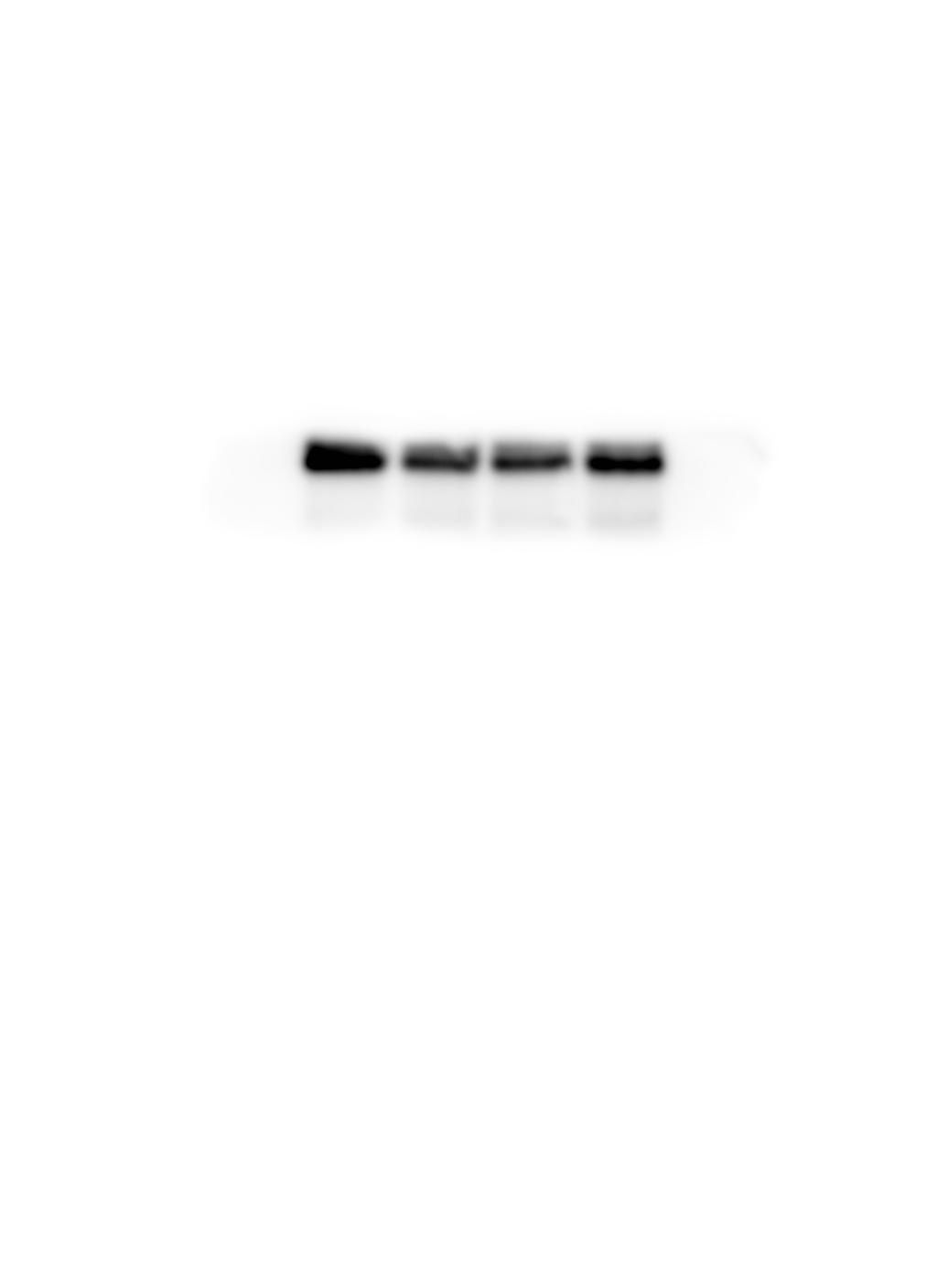

Supplement: Supplementary file 1 [file DataSheet1.zip › raw data/WB/HIF-1 2 .jpg]

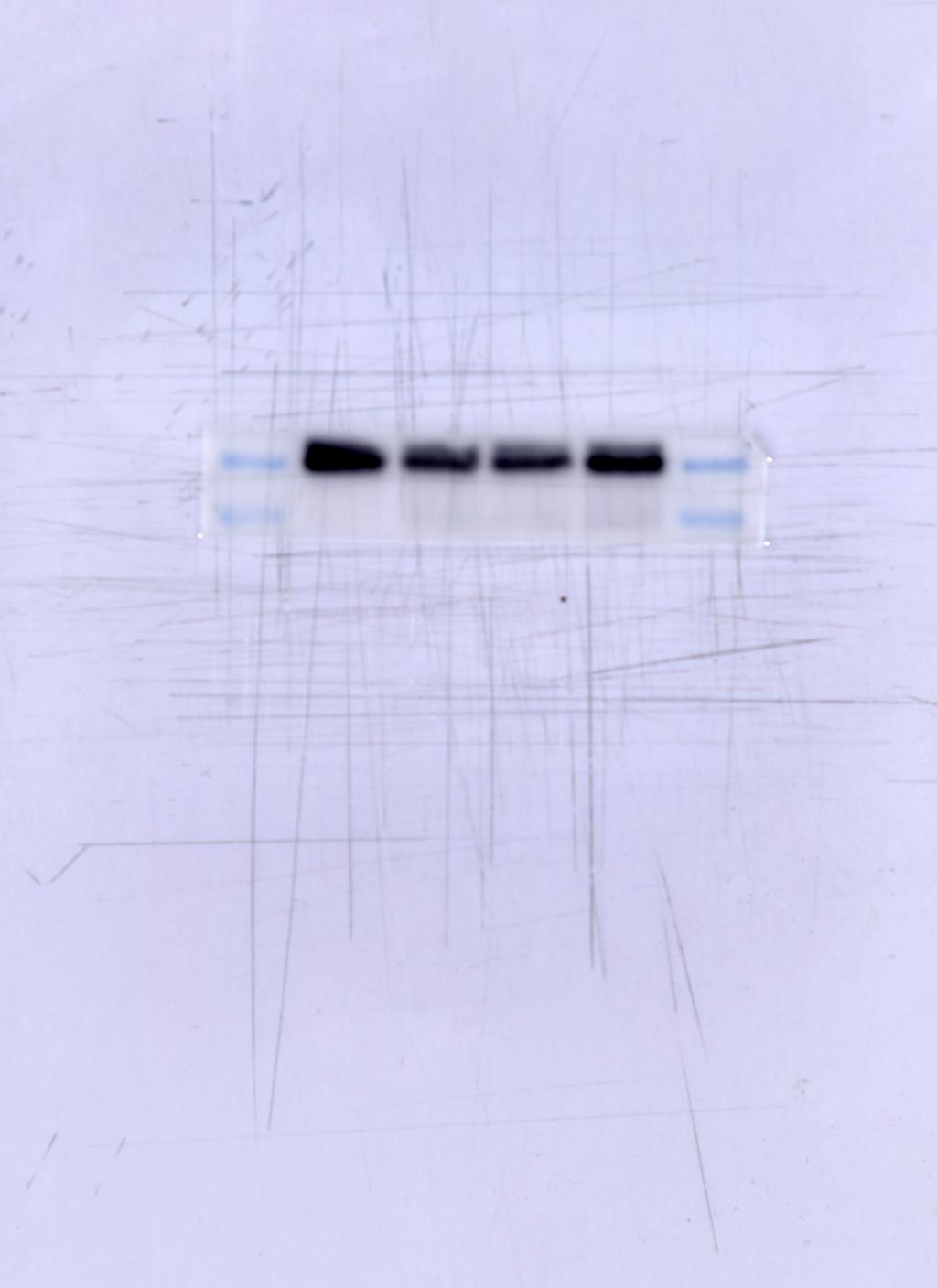

Supplement: Supplementary file 1 [file DataSheet1.zip › raw data/WB/HIF-1 2 marker.jpg]

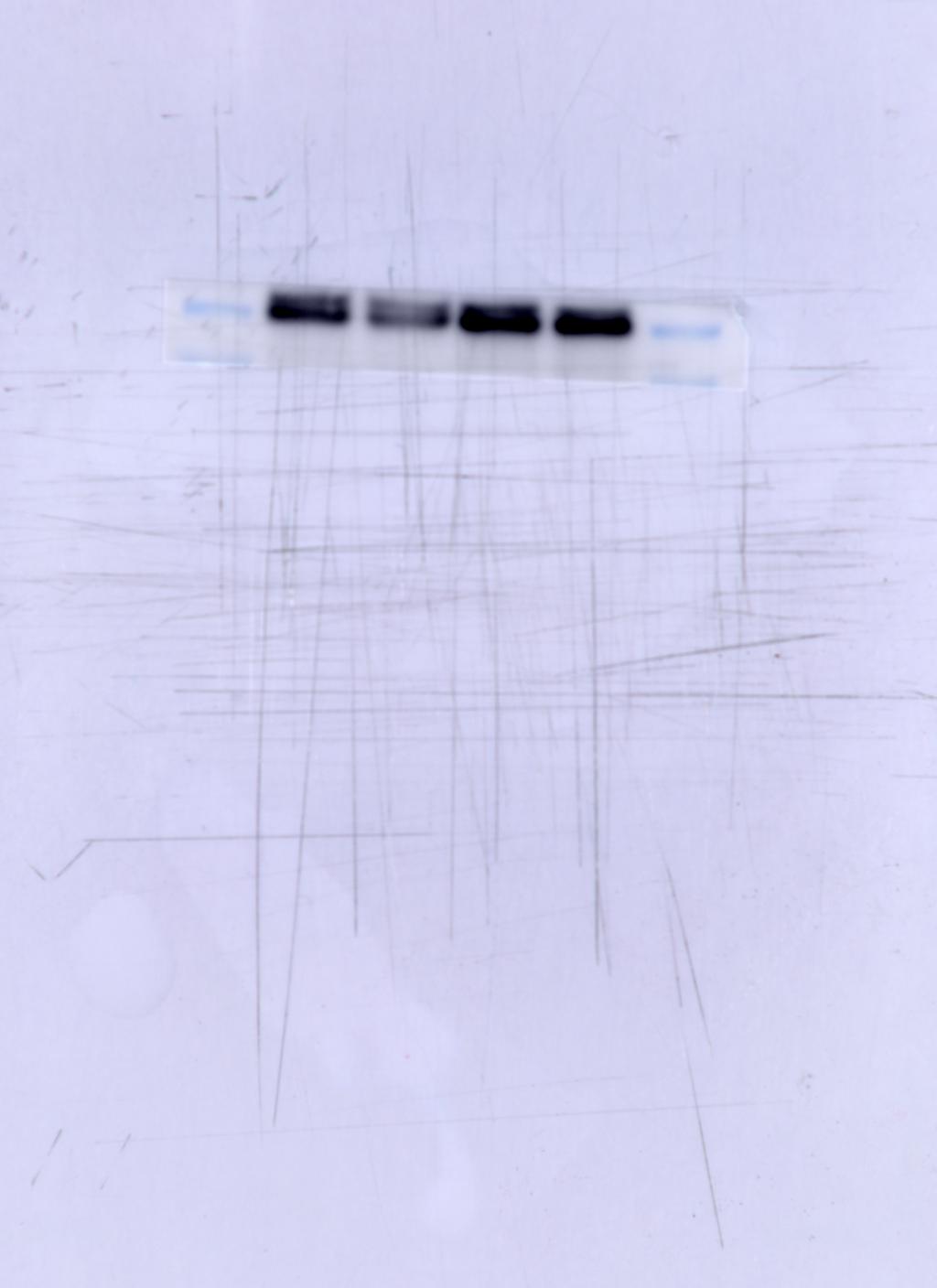

Supplement: Supplementary file 1 [file DataSheet1.zip › raw data/WB/HIF-1 3 marker.jpg]

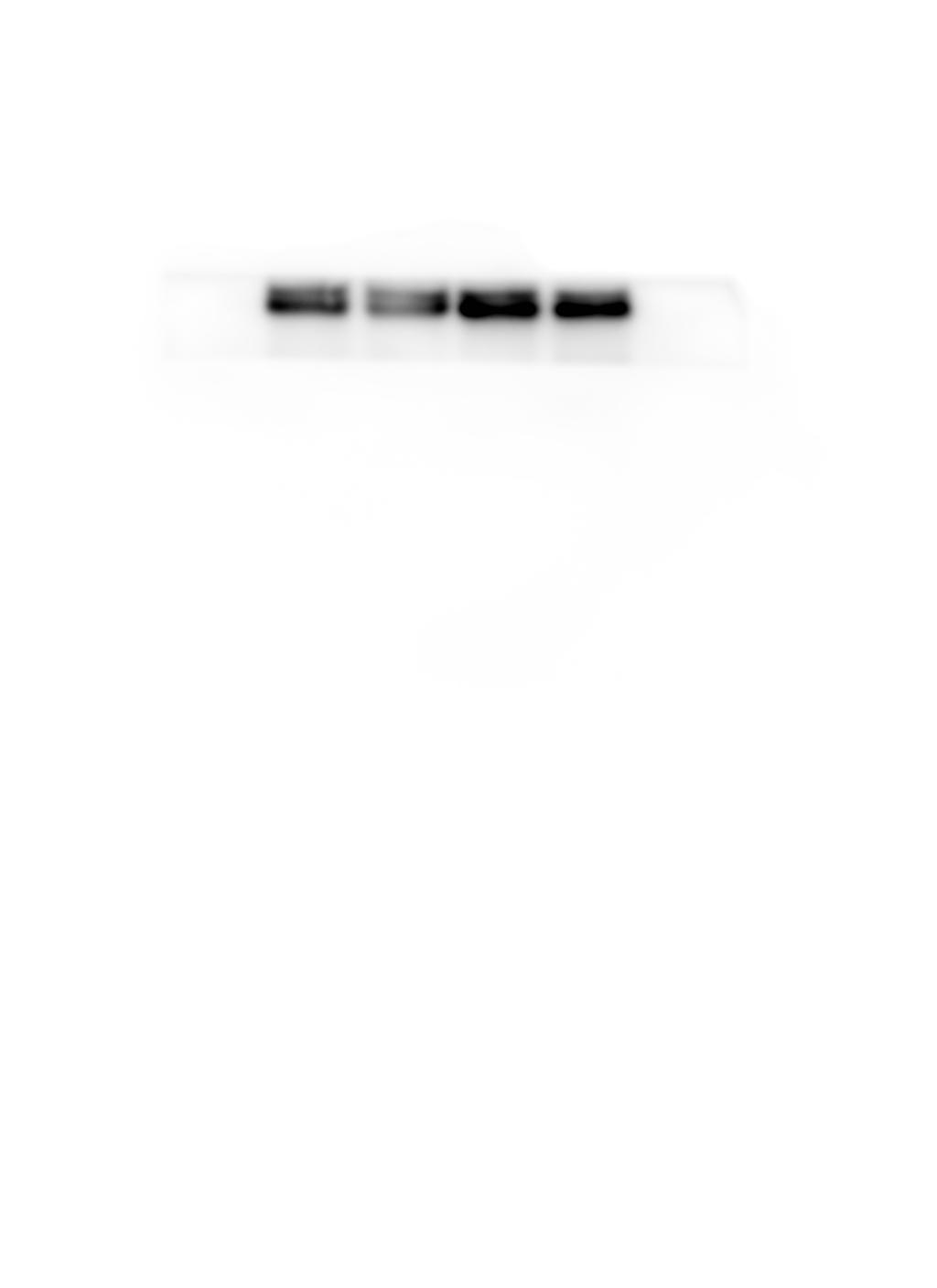

Supplement: Supplementary file 1 [file DataSheet1.zip › raw data/WB/HIF-1 3.jpg]
